# An Overview of Influenza Viruses and Vaccines

**DOI:** 10.3390/vaccines9091032

**Published:** 2021-09-17

**Authors:** Rina Fajri Nuwarda, Abdulsalam Abdullah Alharbi, Veysel Kayser

**Affiliations:** Faculty of Medicine and Health, Sydney Pharmacy School, The University of Sydney, Sydney, NSW 2006, Australia; rnuw0385@uni.sydney.edu.au (R.F.N.); aalh0429@uni.sydney.edu.au (A.A.A.)

**Keywords:** influenza virus, influenza vaccine, neuraminidase, hemagglutinin, vaccine manufacturing, vaccine characterization, recombinant vaccine, universal influenza vaccine

## Abstract

Influenza remains one of the major public health concerns because it causes annual epidemics and can potentially instigate a global pandemic. Numerous countermeasures, including vaccines and antiviral treatments, are in use against seasonal influenza infection; however, their effectiveness has always been discussed due to the ongoing resistance to antivirals and relatively low and unpredictable efficiency of influenza vaccines compared to other vaccines. The growing interest in vaccines as a promising approach to prevent and control influenza may provide alternative vaccine development options with potentially increased efficiency. In addition to currently available inactivated, live-attenuated, and recombinant influenza vaccines on the market, novel platforms such as virus-like particles (VLPs) and nanoparticles, and new vaccine formulations are presently being explored. These platforms provide the opportunity to design influenza vaccines with improved properties to maximize quality, efficacy, and safety. The influenza vaccine manufacturing process is also moving forward with advancements relating to egg- and cell-based production, purification processes, and studies into the physicochemical attributes and vaccine degradation pathways. These will contribute to the design of more stable, optimized vaccine formulations guided by contemporary analytical testing methods and via the implementation of the latest advances in the field.

## 1. The Influenza Virus and Subtypes

The influenza virus is an enveloped virus belonging to the Orthomyxoviridae family. There are four genera of influenza virus that infect vertebrates: influenza virus A, B, C, and D which are distinguished based on antigen differences in their matrix protein and nucleoprotein. Type A is by far the most virulent virus that causes severe respiratory disease or death. It can even lead to the emergence of a new influenza epidemic and a worldwide pandemic. Influenza B viruses also cause the seasonal flu epidemic in humans. There are two circulating influenza B lineages, B/Yamagata and B/Victoria, which are included in the seasonal flu vaccines [1]. Meanwhile, influenza C viruses commonly cause mild symptoms and are not known to cause an epidemic, and influenza D viruses have been recognized to infect swine, cattle, and sheep, and are not identified to cause disease in humans [2,3]. The virus carries a negative-sense, single-stranded RNA (ssRNA) genome that is divided into eight (subtypes A, B) or seven (subtype C, D) separate segments [4,5].

**Figure 1:**
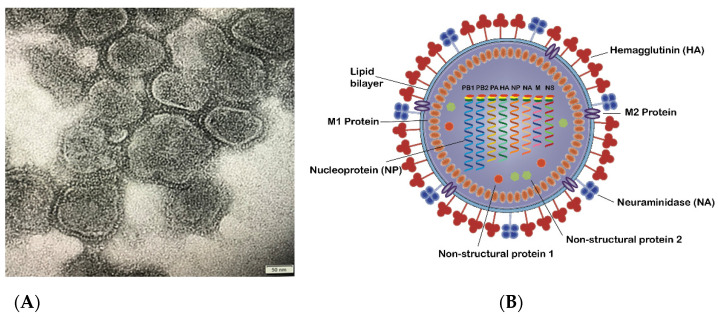
(**A**) TEM image of influenza virus mainly in spherical shapes with particle sizes around 100–150 nm; (**B**) Schematic diagram of an influenza A virus, representing the virus components, including surface glycoproteins HA and NA, M1, and M2 protein, nonstructural proteins, and nucleoproteins.

Influenza virus A and B are indistinguishable in shape under transmission electron microscopy (TEM) (Figure 1). They have spherical or filamentous forms of about 100 nm in diameter for spherical structures and frequently over 300 nm in length for the filamentous shape [6]. The influenza A virion is covered with viral surface glycoproteins of hemagglutinin (HA) and neuraminidase (NA). Each virion with an average size of 120 nm has approximately 300–400 HAs and 40–50 NAs on its lipid membrane, although the numbers of each protein vary between the different subtypes [7,8,9]. These two glycoproteins become the basis of further subdivisions of influenza A virus. To date, 18 subtypes of HA and 11 subtypes of NA have been identified. H1-3 and N1,2 strains (e.g., as in A/H1N1 and A/H3N2) predominantly infect people and are currently circulating as seasonal influenza strains amongst humans. Influenza infection with other subtypes, such as highly pathogenic H5N1, has also been recorded to cause outbreaks in poultry and human infection [10,11,12,13].

A small number of matrix ion channel proteins (M2) across the viral membrane with a ratio of one M2 per 10^1^–10^2^ molecules of HA also exist [14]. The lipid envelope and its three proteins (HA, NA, and M2) coat a matrix protein called M1, which encircles the virion core. The inner part of the virion contains the non-structural protein 2 (NS2) and the ribonucleoprotein (RNP) complex, which comprises the RNA segments layered with the nucleoprotein (NP), and RNA-dependent RNA polymerase consists of two subunits of polymerase basic (PB1 and PB2) and one subunit of polymerase acidic protein (PA).

The configuration of the influenza B virion is almost identical to four membrane proteins: HA, NA, and in place of M2, NB and BM2 are found in the subtype B virus. Influenza C and D virions are structurally distinct from those of the A and B viruses, having only a single primary surface glycoprotein, the HA-esterase-fusion glycoprotein, which has a similar function to HA and NA of subtype A and B viruses. Influenza C and D virus particles have a variety of shapes: they are elliptical or spherical with a diameter of 80–120 nm, or filamentous with a similar diameter but a length in the µm range [15,16,17].

## 2. Influenza Virus Life Cycle

**Figure 2:**
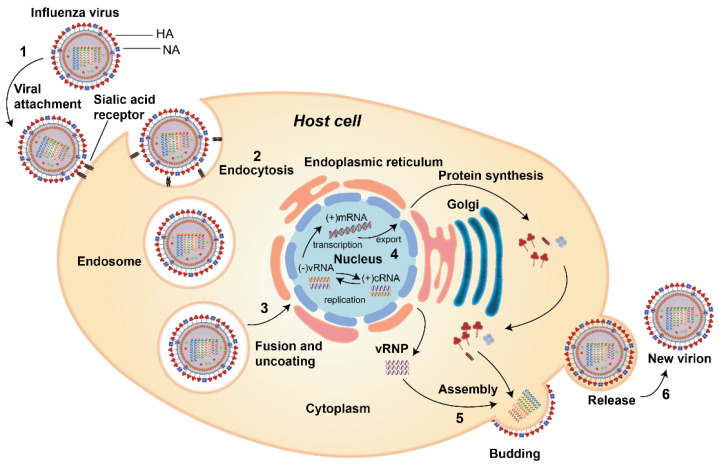
The life cycle of influenza A virus: (1) virus attachment to sialic acid receptor via HA; (2) entry of the virus into the host cell via endocytosis; (3) fusion and uncoating of virus particle; (4) vRNPs entry into the nucleus followed by transcription and replication of the viral RNA genome and then export of vRNPs from the nucleus; (5) assembly of viral components and budding at the host cell membrane; (6) new virion release from the host cell.

The replication cycle of the influenza virus goes through several stages as shown in Figure 2, and is as follows: (1) virus attachment to the sialic acid receptor, (2) entry of the virus into the host cell, (3) fusion and uncoating of the virus particles, (4) vRNPs entry into the nucleus followed by transcription and replication of the viral RNA genome then export of vRNPs from the nucleus, (5) assembly of viral components and budding at the host cell membrane, and finally (6) the release of new virions from the host cells.

(1) Virus attachment to the sialic acid receptor: the first stage of viral infection is the attachment of HA to the sialic acids located at the host cell membrane’s surface. Sialic acids are connected to the carbohydrates of HA via glycosidic linkage. There are two linkages which are essential for the specificity of HA; (i) α (2, 3) linkage which is abundant in the digestive tract in avian or in the bronchial tissue in human, monkeys, horses, as well as upper respiratory tract of the lung epithelium in swine, and (ii) α (2, 6) which is found on the cell surface of the human upper respiratory tract as well as the trachea of bats and swine [4,18].
(2) Entry of the virus into the host cell: receptor-mediated endocytosis occurs upon virus binding, and the virus enters the host cell in an endosome.
(3) Fusion and uncoating of the virus particles: The acidic pH of the endosome (pH 5–6) causes the fusion of the viral and endosomal membranes and opens up the M2 ion channel protein and acidifies the nucleus, thus allowing vRNP to be released from M1 to enter the host cell’s cytoplasm and then to the nucleus.
(4) vRNPs entry into the nucleus followed by transcription and replication: the viral proteins that constitute the vRNP (NP, PA, PB1, and PB2) detect the nuclear localization signals which can attach to the cellular nuclear import machinery and consequently, enter the nucleus to undergo the transcription and replication processes. The negative-sense RNA is first transformed into a positive-sense RNA and serves as a template for the generation of viral RNA, followed by the internal RNA synthesis initiated by the viral RNA-dependent RNA polymerase (RdRp). Through its C-terminal domain, the RdRp associates with the large subunit of RNA Polymerase II (Pol II), which continue the transcription to produce mature mRNA. vRNPs are then exported out from the viral core through the nuclear pores [19,20].
(5) The assembly of virus components and budding: the virus’ protein components, i.e., HA, NA, and M2, are transported to the membrane’s apical region where the virions bud from the polarized epithelial cells.
(6) The release of new virions from the host cells: after forming viral particles, the sialic acid residues from glycoproteins and glycolipids are cleaved by NA, enabling the newly synthesized viral particles to be released from the host membrane and spread to the nearby cells [21].

## 3. HA and NA of Influenza Virus

**Figure 3:**
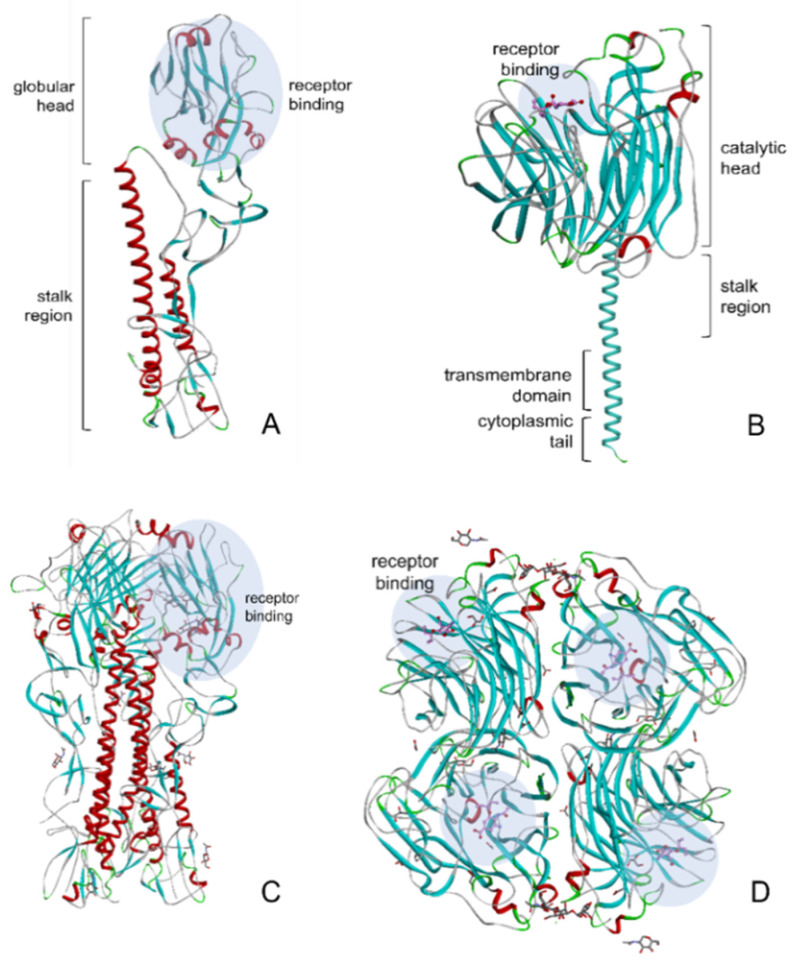
Ribbon diagrams of the 1918 human HA (Protein Data Bank (PDB) ID: 1RUZ) and 2009 H1N1 NA in complexes with oseltamivir (PDB ID: 3TI6) with their receptor binding sites shown in blue circles. (**A**) HA monomer; (**B**) NA monomer; (**C**) trimeric HA and (**D**) tetrameric NA shown from the top.

HA and NA are the major transmembrane glycoproteins, with HA representing roughly 80% of the virus surface. These two glycoproteins recognize the same molecule of the host cell, sialic acid (SA) [22]. In addition to the virus’ infectivity and transmissibility, these two proteins are also associated with virulence properties, host specificity, and resistance to antiviral treatment of the virus [7]. The ribbon diagram of HA and NA structures is shown in Figure 3.

HA comprises three indistinguishable subunits (homotrimer) attached to the viral lipid membrane with each monomer possessing a globular head containing the receptor-binding domain, and a stalk region (Figure 3A) [23]. This protein is responsible for the initial contact between the virus and its target cell containing terminal sialic acid residues. Moreover, HA contributes to the virus’ internalization with the host cell through endocytosis, thus delivering the nucleoprotein into the cytoplasm. HA acts as the major antigen on the flu virus and becomes the primary target for antibody neutralization [24]. 

The NA (Figure 3B) is a tetrameric protein with four identical polypeptides consisting of a globular head, a cytoplasmic tail, a transmembrane domain, and a stalk region [25]. It is an enzyme that destroys the receptors recognized by HA by catalyzing the cleavage of terminal α-(2,3 or 2,6)-ketosidic linkage sialic acid from a range of glycoconjugates such as glycoproteins and glycolipids, therefore playing an essential role in the replication cycle of the virus. NA acts as a biological scissor that cleaves sialic acid residues from HA and cell surface glycans and facilitates the movement of the virus during virus entry, as well as from the surface glycoprotein of newly synthesized virions, hence allowing the virus to be released from the host cell and carry on the infection to other cells [26].

Influenza viruses are completely reliant on a well-balanced action of HA and NA to establish a productive infection. During initial infection, an increase in receptor-binding affinity appears to necessitate an increase in the viral NA’s receptor-destroying activity as well. On the other hand, enhanced receptor binding is a serious disadvantage in the late stage of infection because it prevents new virions from being released from host cells [27].

## 4. Glycosylation of HA and NA

During the replication cycle of the influenza virus, small changes in the virus’ genes via mutations might occur due to the error-prone replication process resulting in antigenic drift (Small changes or mutation in the virus genes which can lead to changes in HA or NA.). Aside from the alteration in amino acid sequences, antigenic drift often involves the changes in the glycosylation patterns of viral glycoproteins. HA and NA both possess potential regions for N-linked glycosylation, the addition of oligosaccharide chains through N-glycosidic bonds to the Asn residues which exist as part of the Asn-AA-Ser/Thr sequence, where AA can represent all amino acids except for proline. The attachment happens with the aid of glycosyltransferases and oligosaccharyltransferase enzymes located in the endoplasmic reticulum (ER), followed by the structural alteration of the oligosaccharides by mannosidases and glycosidases during transfer through ER and Golgi bodies [28]. This post-translational modification is vital for the influenza virus life cycle by affecting the biosynthesis, structural stability, and glycoprotein function.

Glycosylation sites of HA are located in the stalk region and contribute to the molecular stability of HA, its receptor binding and the immune response of the host [29]. Prior studies have also suggested that glycosylation on the HA globular head domain may expose or protect the antigenic sites, leading to virus evading neutralizing antibodies [30,31,32,33,34].

**Figure 4:**
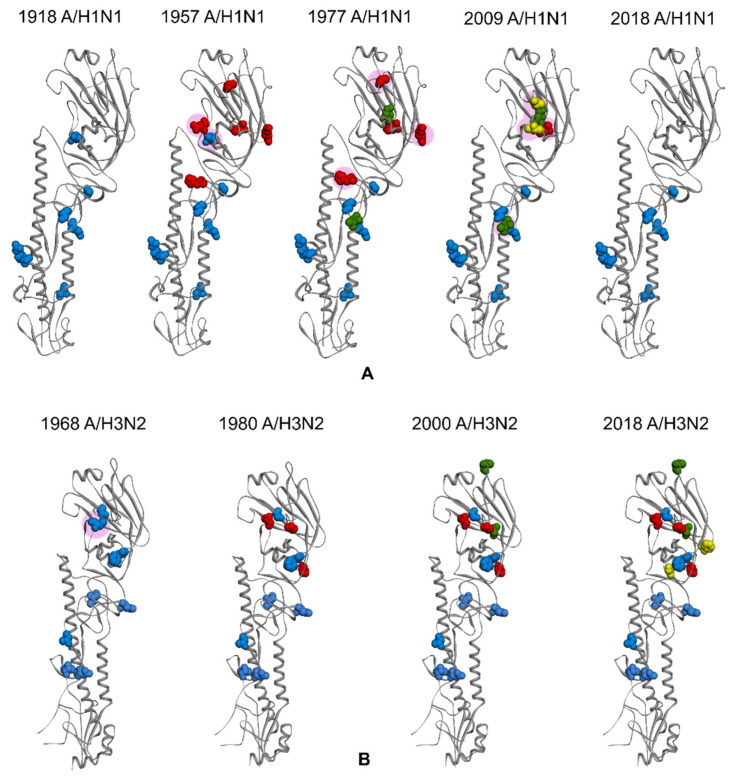
Some of the NLG sites of 1918 HA (**A**) and 1968 HA (**B**). Blue color residues represented the NLG sites when they were first identified, while red, green, and yellow color residues represent NLG sites acquired afterward. The pink circle represents the NLG sites that have been lost during the evolution. The 1918 A/H1N1 initially possessed six NLG sites and acquired five more in 1957. The 1977 A/H1N1 gained two NLG sites but lost two, whereas 2009 A/H1N1 gained two sites and lost three. The current 2018 A/H1N1 circulating strain has lost five more NLG sites compared to the 2009 strain. Meanwhile, NLG sites in 1968 A/H3N2 HA globular head have been gradually increasing from initially two in 1968 to eight in the current 2018 strain (with the loss of site 97 in 1975).

Four seasonal human influenza strains, two A subtypes (A/H1N1 and A/H3N2) and two B lineages (B/Victoria and B/Yamagata) have circulated globally since 1977 [35,36]. Figure 4 demonstrates the evolvement of the glycosylation patterns of A/H1N1 and A/H3N2 strains from 1918 to 2018, i.e., when they were first introduced to humans and those currently circulating. The glycosylation in the stalk region of HA of the 1918 pandemic A/H1N1 and 1968 A/H3N2 influenza viruses were known to be highly conserved. The 1918 A/H1N1 possessed six conserved N-linked glycosylation (NLG) sites at residues 27, 28, 40, 304, 498, and 557 (Figure 4A), while 1968 A/H3N2 had five glycosylation sites at residues 24, 38, 54, 301, and 499 (Figure 4B). The globular head of HA, on the other hand, had gone through subsequent glycan gains and losses over the years. The number of NLG sites in the A/H3N2 HA globular head has increased from initially two (at residues 97 and 181) in 1968, to eight (at residues 79, 138, 142, 149, 160, 174, 181, and 262) on the current 2018 seasonal strains, after the disappearance of site 97 around 1975. Similarly, 1918 A/H1N1 acquired an increasing number of glycans until its disappearance in 1957 and had the same glycosylation profile when it re-emerged in 1977 until the 2009 A/H1N1 strain replaced it. The currently circulating A/H1N1 strain is the result of a reassortment of swine lineage viruses and the glycosylation is similar to the very early human A/H1N1. Meanwhile, the NLG sites for the influenza B virus have been relatively stable at around 10–12 sites since the splitting of Victoria and Yamagata lineages in 1983 [28].

Changes in glycosylation sites and glycan profiles in HA affect how viruses interact with the host immune system. The evasion of neutralizing antibody recognition enables the virus to continue circulating in the human population. Consequently, more studies regarding the glycosylation pattern of viral glycoprotein are needed to design better vaccines against influenza viruses [34,37].

While the NLG of HA is known to contribute to immune escape and influenza viruses’ virulence, it is still unclear how the NLG of NA affects its function. Previously published research established that the glycosylation of the N8 NA protein is necessary for proper tetramerization [38]. Additionally, glycosylation of the head domain of the NA protein is required for proper maturation [39]. In a recent study, Bao et al., explored the function of the NLG modification in the NA protein of the A/1933/WSN/H1N1 (WSN) virus. The results indicated that the NLG modification of the NA protein was critical for its budding. In transfected cells, non glycosylated NA protein lost its ability to bud. Additionally, the absence of the NLG modification significantly reduced the mutant virus’s budding and replication. The deletion of NLG at the 219 position significantly attenuated the WSN virus in a mouse model. It was suggested that overexpression of non glycosylated NA protein in cells induced the unfolded protein response (UPR), which may affect the ability of NA proteins to bud, thereby attenuating the mutant virus’s virulence [40].

## 5. The Influenza Epidemic vs. Pandemic

Influenza viruses can cause severe contagious disease by infecting the respiratory tract and may result in hospitalization and even death. Annual seasonal epidemics of influenza were predicted to have approximately 3 to 5 million cases of severe illnesses and 290,000 to 650,000 related respiratory deaths globally [41]. The highest mortality rates were estimated in Southeast Asia and sub-Saharan Africa, and the high-risk groups for severe outcome and death include people aged 75 years or older, children younger than five years, and individuals with lingering illnesses that put them at more significant risk of acquiring influenza or its complications [41,42].

New strains of influenza A virus appear sporadically every 2 to 5 years as epidemics and last between 3 and 6 months depending on the region. The influenza epidemic is often referred to as the flu season, which is potentially tied to the weather and climate. The introduction of specific mutations within surface glycoproteins HA and NA enables the virus to evade the host immune system and results in no permanent immunity against the virus after neither natural infection nor vaccination. The minor changes of the influenza virus’ antigenicity are described as antigenic drift and become the basis for the influenza epidemic’s frequent occurrence, making it necessary to update influenza vaccines annually to match the currently circulating strains subject to WHO recommendation [43,44,45]. 

Influenza pandemics, on the other hand, result from significant changes in the virus’s antigenicity and are suggested to happen approximately every 10 to 40 years [46]; however, it could happen at any time. History documented that no less than three pandemics occurred during the twentieth century: the 1918–1919 Spanish flu, 1957–1958 Asian Flu and 1968–1969 Hong Kong Flu, with the respective mortality rates varying from devastating to mild [47]. These pandemic viruses obtained new subtypes by the reassortment of RNA segments from distinct influenza viral strains. This genetic change, referred to as an antigenic shift (An abrupt change in the antigenicity of a virus caused by the recombination of two viral strains’ genomes), permits a viral strain to jump from one species of animal to another and from animals to human [48,49].

The 1918 pandemic, also termed as Spanish Flu, was the first and remains the most disastrous influenza pandemic in history. It was estimated that the virus spread worldwide by infecting about 25–30% of people worldwide and killing approximately 50 million individuals, around 2.7% of the world’s population [50,51,52]. A more recent analysis by Spreeuwenberg et al. in 2018 indicated that earlier estimations were excessively high. Their own estimate puts the death toll at 17.4 million, 0.95% of the world’s population [53]. The 1918 pandemic was caused by the A/H1N1-subtype influenza virus, which was considered as the common ancestor of nearly all human influenza A cases. These include not only antigenically “drifted” descendants of the 1918 H1N1 virus, but also the genetically reassorted pandemic viruses that emerged in 1957 (H2N2), 1968 (H3N2), and 2009 (H1N1) [54]. The H2N2 subtype of influenza A virus which emerged in the Hunan Province of China in February 1957, was believed to be clinically milder than the H1N1 virus that caused the 1918 pandemic with a mortality rate worldwide of 1.1 million deaths [55]. The virus circulated globally until H3N2 appeared in 1968, causing another pandemic. It first emerged in China, then spread rapidly to Hong Kong. The 1968 pandemic, also called Hong Kong flu, was comparatively a milder pandemic with the death toll around 1 million people [56,57]. The influenza pandemic in the twenty-first century started in early 2009 when a novel swine-origin flu virus appeared and substituted the former seasonal influenza H1N1. Initially identified in Mexico, the virus spread rapidly worldwide and was declared a pandemic by WHO in June 2009 after 74 countries reported the infections [58,59]. The virus, also called “2009 H1N1”, was a reassortment product between two pre-existing swine flu viruses—a North American H1N2 and a Eurasian H1N1 [60].

There has been an increase in reports of direct bird-to-human transmission of avian influenza viruses in the last two decades, resulting in an ongoing outbreak of influenza A/H5N1 among poultry with associated human infections. In 1997, fatal cases of H5N1 infections in humans were first detected in Hong Kong. Six years later in 2003, the virus re-emerged as a highly pathogenic avian influenza virus and caused devastating outbreaks in local poultry, raising concerns about a potential influenza pandemic [61,62,63]. The outbreaks had become endemic in several countries, with the highest number of cases occurring in Egypt, Indonesia, and Vietnam [64]. In 2007, there was evidence of limited person-to-person transmission of the H5N1 in China. Furthermore, WHO reported that limited, nonsustained human-to-human H5N1 cases were observed in Indonesia and Pakistan in 2006 and 2007, respectively. The first case of H5N1 infection in the Americas was reported in Canada on January 8, 2014, in a traveler returning from China. Although human infections with this virus are uncommon, approximately 60% of cases result in death [65]. In November 2019, the WHO declared the total number of 861 verified human cases for avian influenza A (H5N1), leading to 455 death cases since 2003, with one last case reported in Nepal on 30 April 2019 [66].

## 6. Vaccines against Influenza Epidemic and Pandemic

Several vaccines exist to combat the influenza epidemics and pandemics. Influenza vaccines are reformulated each year based on global surveillance of the current circulating strain. Therefore, annual vaccination remains the most effective way to prevent seasonal epidemic influenza.

Currently, there are three types of vaccines used worldwide: inactivated influenza vaccine (IIV), live attenuated influenza vaccine (LAIV), and recombinant HA vaccine (Table 1). Each type of vaccine has its advantages and disadvantages. Vaccines can induce immune effectors, including humoral effectors (antibodies produced by B lymphocyte), that can bind specifically to the virus antigen, and cellular effectors (cytotoxic CD8+ T lymphocytes) that can limit the spread of the virus inside the body by killing the infected cells. All influenza vaccines must be annually updated because the immunity only lasts for a short period of time [67] and to match the antigenicity of the circulating viruses [68,69]. Hence, WHO and other stakeholders meet biannually to select the most appropriate influenza viruses from globally circulating viruses according to their antigenic and genetic characteristics and the epidemiologic information from different countries. One of these influenza vaccine composition meetings occurs for the Northern hemisphere and the other for the Southern hemisphere [70,71].

Trivalent vaccines containing two influenza A (H1N1 and H3N2) and one lineage of influenza B viruses were formulated to provide protection against three distinct influenza viruses for many years, even though there are two different lineages of circulating B viruses. Therefore, to give a wider protection against the current circulating viruses, a second lineage of B virus was added to the formulation for a quadrivalent influenza vaccine. Current approved influenza vaccines are quantitatively standardized in terms of HA quantity or antigenicity, but not in terms of neutralizing antibodies (NA). As a result, both the NA content and the NA immunogenicity of vaccinations may be very varied.

### 6.1. Inactivated Influenza Vaccine (IIV)

**Figure 5:**
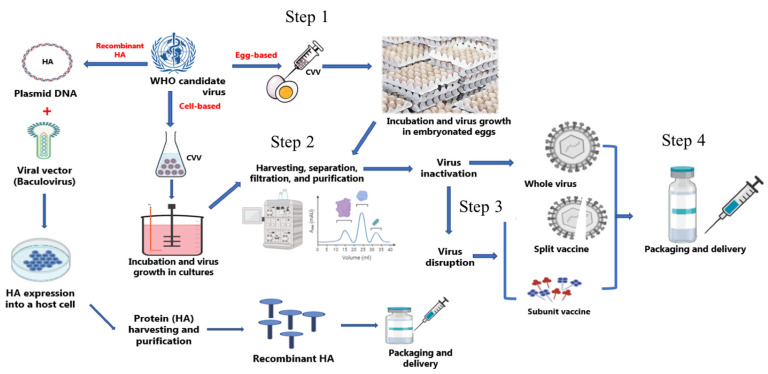
Influenza vaccine manufacturing processes. Both egg-based and cell-based vaccine manufacturing begins with candidate vaccine viruses (CVV) grown in eggs or cells (Step 1). After harvesting, separation, filtration, and purification (Step 2), the virus is inactivated chemically generally using formalin to make inactivated influenza vaccines or disrupted chemically by surfactants to prepare split virus vaccines or subunit vaccines (Step 3). The final step involves fill-finish processes and packaging, delivering, and storage of the product under cold-chain conditions (Step 4). The recombinant influenza vaccine does not need the growth of the whole virus; only the genetic information of the HA antigen is obtained and combined with a baculovirus to facilitate delivery of the genetic information (DNA) into a host cell for expressing the HA antigen. This antigen is produced, harvested, filtered, purified, and then vaccine is prepared.

The inactivated virus vaccine is the most common worldwide approach due to its high safety and relatively low production costs. As a result, it has the highest percentage in the flu vaccine global market. The IIV can be given for children six months and older like Afluria Quadrivalent, Fluarix Quadrivalent, FluLaval Quadrivalent, and Fluzone Quadrivalent; however, some IIVs are only for individuals who 65 years and older, like Fluad and Fluzone High-dose Quadrivalent (Table 1). The virus is generally grown in embryonated chicken eggs or cultured cells of mammalian origin for this type of vaccine (Figure 5). It has been shown that IIV can induce local and systemic immunity, although booster vaccinations might be needed to maintain the antibody titers [85]. IIV has three types: whole-virus inactivated vaccines, split-virus inactivated vaccines, and subunit inactivated vaccines.

#### 6.1.1. Whole-Virus Inactivated Vaccines (WIV)

The whole-virus vaccine was the first of the influenza vaccines to be used. However, a number of systemic and local side effects were reported upon administration. These adverse effects could result from the impurities in these vaccines, such as egg proteins [86]. However, improved technologies in vaccine production have led to safer vaccines with lower impurities and fewer side effects [87]. In WIV, after the incubation and growth in embryonated eggs, virions are chemically inactivated, mainly with formaldehyde or β-propiolactone, then purified (Figure 5). Although this method is the most used approach for influenza virus inactivation, other methods can be used for virus inactivation, including heat and radiation [88]. 

The use of this type of influenza vaccine is currently limited due to the other types that have entered to the market, such as split-virus inactivated vaccines, which have fewer reactogenic effects. The vaccine 3Fluart is a WIV produced by Fluart Innovative Vaccines Kft. and is used only in Europe.

#### 6.1.2. Split-Virus Inactivated Vaccines

The majority of the currently used influenza vaccines are split-virus or subunit vaccines due to their adequate immunogenicity and ease of production. However, they lose some of the inherent immunogenicity because of the lack of the whole virus structure, unlike WIV [87]. The split-virus vaccine can be prepared by disrupting the virus membrane chemically using surfactants such as Triton-100 or Octyl glycoside [89]. Then, the surfactant is removed by tangential flow filtration. The seasonal influenza split-virus vaccines in the US (representing the northern hemisphere) and Australia (representing the southern hemisphere) are summarized in Table 1 with their manufacturer and age group information. In Australia, most of the seasonal influenza vaccines are split-virus inactivated vaccines with five vaccines in the market (FluQuadri (by Sanofi, Pennsylvania, USA), Vaxigrip Tetra (by Sanofi-aventis, Auck-land, New Zeland), Fluarix Tetra (by GlaxoSmithKline Biologicals, Dresden, Germany), Afluria Quadrivalent (by Seqirus, Melbourne, Australia), and Influvac Tetra (by Abbott, Hoofddorp, Netherlands)). In US, also split-virus inactivated vaccines have the dominant part of seasonal influenza vaccines with five (Afluria Quadrivalent (by Seqirus, Melbourne, Australia), Fluarix Quadrivalent (by GlaxoSmithKline Biologicals, Dresden, Germany), FluLaval Quadrivalent (by ID Bio-medical, Laval, QC, Canada), Fluzone Highdose Quadrivalent (by Sanofi, Pennsylvania, USA), and Fluzone Quadrivalent (by Sanofi, Paris, France)).

#### 6.1.3. Subunit Inactivated Vaccines

Generally, subunit vaccines contain only the antigenic parts of the virus: HA and NA. These proteins are either purified from the influenza virus after splitting the virus using surfactants (Figure 5). This type of influenza vaccine usually needs an adjuvant to induce an adequate immunogenicity, especially in the elderly [90].

Fluad and Fluad Quadrivalent are subunit influenza vaccines by Seqirus that can be given to 65 years and over. Another vaccine, Influvac Tetra manufactured by Mylan Health, is used for three years and older (Table 1). Due to their comparable immunogenicity and low reactogenicity, split-virus and subunit-virus vaccines have been more commonly used than whole-virus vaccines since the 1970s [91].

### 6.2. Live Attenuated Influenza Vaccines (LAIV)

The LAIV was developed to mimic natural infection and immunity without causing severe illness and consequently induces humoral and cellular immunity. LAIV was first proposed back in the 1960s by growing the influenza viruses under suboptimal conditions in eggs [92].

These attenuated viruses are temperature-sensitive and grow only at 25 °C (cold-adapted). Cold-adapted donor viruses undergo several passages with a gradual reduction in the temperature in embryonated chicken eggs. Thus, LAIV can grow at the same temperature range as the nasopharynx’s mucosal surface when LAIV is administered intranasally [93]. LAIV has some superiority over IIV because it induces mainly local mucosal immunity and local immunoglobulin A (IgA) production as well as IgG [85,93,94], in contrast to only systemic IgA and IgG antibodies with IIV. However, due to the risk of using live viruses for immunization, LAIV is not recommended for immunocompromised individuals with low immunity (immunocompromised) or people who are in close contact with them.

FluMist Quadrivalent (Table 1), which is manufactured by MedImmune and approved in the US, is an intranasal LAIV seasonal influenza vaccine that can be used safely in children and adults, ages 2 to 49 years, and contain four influenza strains (two influenza A and two influenza B). [95,96,97]. Fluenz Tetra, manufactured by AstraZeneca and approved in Europe, is another LAIV used for ages from 2 to 17 years. No LAIV is approved in Australia as of 2021.

### 6.3. Recombinant HA Vaccine

Recombinant HA vaccine can be produced using recombinant protein expression technology by insect cells and baculovirus due to their high yield and cost effectiveness (Figure 5) [98]. Insect cells are the typical cell lines used for recombinant protein expression, and the Gibco Sf9 cell line is the most commonly used in human or veterinary medicine for recombinant protein expression [99]. 

The recombinant HA vaccine has an advantage over egg-based influenza vaccines: there are no unwanted mutations from egg adaptation (Table 1). Moreover, recombinant HA vaccine is an appropriate option for people with egg allergy [100]. Although recombinant HA vaccine and IIV have a similar mechanism of action, recombinant HA vaccine is less immunogenic and hence requires three times more HA than IIV in order to induce the same antibody titers as in IIV [101]. Another advantage of the recombinant HA vaccine is its suitability for an influenza pandemic because it might take a shorter time to manufacture, and it would be much safer than other influenza vaccines due to the absence of highly pathogenic viruses [102]. However, due to its lack of efficacy in children, the recombinant HA vaccine is limited to adults [69,103]. Flublok Quadrivalent, manufactured by Sanofi Pasteur, is the first recombinant HA vaccine (Table 1).

## 7. Influenza Vaccine Manufacturing Processes

At present, influenza vaccines can be produced by three different approaches: conventional egg-based vaccines (the traditional method), cell-based vaccines, and recombinant HA vaccines. These methods are summarized in Figure 5. Both egg-based and cell-based vaccine manufacturing begins with candidate vaccine viruses (CVV) grown in eggs or cells (Step 1). After harvesting, separation, filtration, and purification (Step 2), the virus is inactivated chemically, generally using formalin, to make inactivated influenza vaccines, or disrupted chemically by surfactants to prepare split virus vaccines or subunit vaccines (Step 3). The recombinant influenza vaccine does not need the growth of the whole virus; only the genetic information of the HA antigen is obtained and combined with a baculovirus to facilitate the delivery of the genetic information (DNA) into a host cell for expressing the HA antigen. The final step involves fill-finish processes and packaging, delivering, and storage of the product under cold-chain conditions (Step 4).

### 7.1. Egg-Based Vaccines

Since the 1940s, egg-based vaccines have been the most common method for producing millions of influenza vaccines annually [104,105]. An abundant egg supply is needed to grow target viruses in chicken eggs regardless of vaccine strain [106]. This method starts with the selection of the predicted circulating virus strains, followed by the reassortment of the genetic segments of HA and NA in an egg-adapted virus that contains other gene segments from strains that can grow in the eggs in large amounts with a good safety profile, such as A/Puerto Rico/8/1934 [103]. After these reassorted viruses are generated, they are injected in embryonated chicken eggs to grow and then are sequenced for confirmation to collect the candidate virus. The approved virus is then delivered by centers affiliated with WHO Global Influenza Surveillance and Response System (GISRS) to private manufacturers [107]. The manufacturers mass-produce the virus in embryonated chicken eggs, then purify the reassorted viruses. Then, the purified viruses are chemically inactivated using formalin or β-propiolactone, and the content of HA is standardized by removing the contaminants of nonviral proteins [108]. The HA protein is the key factor for the induction of antibodies against influenza viruses and, consequently, is the focus of influenza vaccine development [109,110].

Although the egg-based manufacturing method has many benefits, such as obtaining generally a high titer of influenza virus and significant experience in large-scale production, several drawbacks can affect its efficiency. Firstly, the adaptation of the reassortant viruses in eggs and screening the antigenicity of isolate viruses delay the time needed for the production of influenza vaccines for the next influenza season and, consequently, necessitate the start of production of influenza vaccines long before the upcoming influenza season [111,112]. Some influenza strains (like H3N2 viruses), however, are poorly grown in eggs [113]. As a result, the production of influenza vaccines with such strains takes a long time and delays the delivery of the vaccines by affecting the standard production time frame [114]. During egg-based manufacturing, viruses are not monitored and mutate. This egg adaptation can lead to the production of viruses that poorly match the circulating strains, which contributes to the decrease in vaccine efficacy [113,115]. Additionally, there is a biosecurity concern due to the potential risk of exposure to the avian influenza virus in flock houses.

### 7.2. Cell-Based Vaccines

The FDA announced the approval of Flucelvax in 2012, the first approved cell-based influenza vaccine in the US. Madin−Darby canine kidney (MDCK) cells are used to grow viruses [116]. Reassortment of viruses in the cell-based method can be generated similar to the egg-based method using the HA and NA of selected strains [105]. The cell-based approach has some advantages over the egg-based method. Firstly, using cells can overcome the limitation of egg-shortage and offers a more flexible vaccine production approach due to the dependence on the bioreactor’s capacity. Thus, cell-based vaccines can be produced in less time compared to egg-based ones [105]. Moreover, a more heterogeneous glycosylation profile of HA protein can be observed when growing the virus in egg [117]. Some studies investigate the theory that using mammalian cells as a substrate can enhance the vaccines’ immunogenicity compared to avian cells [118].

Furthermore, cell-based vaccines are a suitable alternative for people who have previously experienced egg allergy, as their products are free from egg protein. Interestingly, one of the most important advantages of the cell-based method is the lack of HA mutations due to egg adaptation. Thus, in 2016, the FDA has approved the use of cell-based virus to prevent further mutations caused by egg adaptation [119].

Although advantages of the cell-based method exist, some drawbacks remain. Experience in large-scale production of cell-based vaccines is still less than that of egg-based vaccines. Moreover, according to the CDC, Flucelvax can cost 40% higher than egg-based vaccines. Albeit to a lesser degree, mutations can still happen in HA and NA proteins in cell culture due to serial passaging [120].

## 8. Influenza Vaccine Formulation and Ingredients

Vaccines must contain ingredients that are confirmed to be safe for humans. In addition, each vaccine component serves a specific purpose, i.e., increasing the immunogenicity of antigens and keeping the vaccine formulation stable.

### 8.1. Active Components

The viral antigens, the active pharmaceutical ingredient (API) serves as the immunogenic component that is the most crucial part of the vaccine. Every influenza season, WHO has recommended four influenza viruses to be included in quadrivalent vaccines and three strains in trivalent vaccines. Thus, quadrivalent influenza vaccines contain four influenza strains (two influenza A strains and two influenza B strains), and trivalent influenza vaccines contain three influenza strains (two influenza A strains and one influenza B strain).

### 8.2. Adjuvants

Adjuvants are compounds that improve the immune reaction to a vaccine. Aluminum salts such as aluminum hydroxide (Panflu), aluminum phosphate, and potassium aluminum sulfate are among them. Adjuvants are believed to increase the immune response by having the antigen(s) close to the injection site, where immune system cells can easily reach them [121]. The usage of aluminum adjuvants in vaccines usually results in less antigen per dose of vaccine and, in certain instances, requires fewer vaccine doses. The involvement of adjuvants in vaccines is often linked to local reactions at the injection site following vaccination [122]. Other adjuvants can be used in flu vaccines such as virosomes or oil-in-water emulsions (MF59, AS03, AF03). Fluad Quad is the only adjuvanted (MF59) flu vaccine in Australia.

### 8.3. Stabilizers

Stabilizers are used to preserve a vaccine’s potency by maintaining the antigen and other vaccine ingredients intact during storage. Stabilizers also keep vaccine ingredients from sticking to the side of the vaccine vial. Examples of stabilizers include sugars (lactose and sucrose) and amino acids (glycine and monosodium glutamate) [123].

### 8.4. Preservatives

Preservatives are used in some vaccine formulations to eliminate fungal and/or bacterial infections in vaccines. Preservatives were initially introduced to avoid bacterial contamination in multidose vials (not used in individual vial vaccines). Thiomersal, phenoxyethanol, and phenol are among the preservatives utilized. Preservatives have been used in numerous vaccinations, although there have been relatively few significant harmful effects linked with their use worldwide, such as allergy [124].

### 8.5. Trace Components

Trace components in vaccines are the minute amounts of chemicals or substances used in the early stages of the manufacturing, or leachables, e.g., from the container. Depending on the production procedure, trace quantities of cell culture fluids, egg proteins, antibiotics, or inactivating agents can be present [124]. In certain instances, only small amounts of these compounds are found in the final vaccine component.

Antibiotics are occasionally utilized during the processing phase to guarantee that contamination by bacteria does not arise. Some influenza vaccines contain antibiotics such as Neomycin, Polymyxin B, and/or Gentamicin. Moreover, prefilled vaccine syringes may contain traces of silicon oil that is used as a lubricant in prefilled vaccine syringes.

During the development of WIV and split-virus inactivated vaccines, inactivating agents are used, and the final vaccine contains relatively few of these inactivating chemicals or splitting agents, such as formaldehyde, glutaraldehyde, or surfactants, respectively [88].

## 9. Novel Influenza Vaccine Platforms

### 9.1. Virus-Like Particle (VLP) Vaccines

VLPs are composed of surface viral glycoproteins that self-assemble to form nonreplicating particles that look like viruses. These particles mimic the native viral structure but lack genetic materials and cannot cause an infection [125]. VLPs were successfully developed as vaccines and have been used against several viruses, including hepatitis B virus (Heptavax-B (by Merck, Whitehouse, NJ, USA), Engerix-B (by GlaxoSmithKline Biologi-cals, Rixensart, Belgium), H-B-VaxII (by Merck, Whitehouse, USA), and Hepavax-Gene (by Janssen Vaccines Corp., Incheon, South Korea), human papillomavirus (Gardasil (by Merck, Whitehouse, USA), Cervarix (by GlaxoSmithKline Biologicals, Rixensart, Belgium), Cecolin (by Wantai Biopharma, Beijing, China), and Gardasil-9 (by Merck, Whitehouse, USA)), and hepatitis E virus HEV (Hecolin, by Wantai Biopharma, Beijing, China) [126]. Therefore, the VLP platform has proven to be a good strategy to produce vaccines including for influenza. VLPs have been combined with hepatitis B virus core (HBc), and a recombinant M2 protein containing three tandem copies of M2e (3M2e), as well as nucleoprotein (NP) epitopes to overcome the challenge of rapid influenza evolution [127]. Innate immunity can be activated by VLPs directly or by stimulating antigen-presenting macrophages or dendritic cells, which can lead to the efficient induction of virus-specific T- and B-cell responses. Moreover, influenza VLP vaccines can contain several different viral proteins (HA, NA, M1, or M2e) in combination or individually on single VLPs, providing some flexibility in vaccine design for broader protection. The addition of pure viral neuraminidase to a traditional influenza vaccination resulted in a balanced and wide immune response [128]. Moreover, because of the protein’s conserved nature, M2e is a potential target for universal vaccine development. Cross-reactive antibody responses and significant protection levels against homologous and heterologous A subtypes were induced by the VLPs composite of M1 and NA [129]. Some of the VLP influenza vaccine candidates have reached clinical trials (Table 2). A recombinant A (H1N1) 2009 influenza VLP vaccine (HA) by Novavax was evaluated in phase 2 clinical trials in 2012 for safety and immunogenicity [130]. This VLP influenza vaccine showed a robust immune response after a single vaccination, with only mild adverse events. Moreover, a plant-based QVLP (HA) by Medicago completed phase 2 clinical trials in 2019 [131]. The plant-based VLP influenza vaccine was able to induce robust antibody responses and antigen-specific CD4+ T cells. These VLP vaccine candidates were able to produce humoral and/or cellular responses. It also showed cross-protection by inducing antibodies against four different influenza strains (two influenza A and two influenza B) [131].

### 9.2. Antigen-Presenting Cell (APC) Inducible Vaccines

CD11c+ dendritic cells (DCs) contribute significantly to adaptive antiviral immune responses during viral infection by accumulating and converting viral antigens into peptides for presentation to T-cells in secondary lymphoid organs through the main histocompatibility complex (MHC) [138].

Some preclinical work has been done to strengthen the T-cell response to the influenza virus by inducing the antigen-presenting cells. For instance, Fonteneau and colleagues have studied the in vitro impact of influenza virus infection on blood-purified DCs and their capacity to accumulate and present viral antigens to CD8+ and CD4+ T-cells. They demonstrated that exposure to influenza virus induces an expansion of anti-influenza virus T helper 1 (TH1) CD4+ T-cells and cytotoxic T lymphocytes CTLs. These findings indicate a novel function for DCs in the generation of antiviral T-cell responses, suggesting that these DCs play an important part in the adaptive immune response to viruses [139]. Another approach has shown that grafting the alpha-Gal epitope onto HA enhances the APCs’ influenza vaccine virus uptake [140]. Thus, immunogenicity of influenza virus vaccines may be enhanced by efficiently targeting vaccinations to APCs. Additionally, influenza HA can be targeted to APCs using fusion DNA vaccines which encode proteins that bind to influenza HA bivalently to target them to distinct surface molecules on APCs [141]. This targeting is able to induce the immune response toward either CD8+/Th1 T cell response or antibody/Th2 response [141].

### 9.3. Nanoparticle-Based Influenza Vaccines

Conventional influenza vaccines can provide systemic protection against the virus; however, local protection in the mucosal respiratory is also desirable against the influenza virus [142,143]. Nanoparticles have characteristics such as solubility and stability that make them suitable for mucosal immunity as they induce immunity that works against respiratory viruses. These nanoparticles can be derived from a natural or synthetic source and work as antigen carriers [144]. Thus, more studies have been done using nanoparticles against the influenza virus than other viruses. Papaya mosaic virus nanoparticle was used as an adjuvant with trivalent inactivated flu vaccines, and it has been tested to immunize mice. This nanoparticle vaccine was found to be superior to IIV in terms of induction of anti-influenza immunity based on IgG2, IgA, and IgG titers in bronchoalveolar lavage samples and serum, especially after intranasal immunization [145].

Moreover, a polylactic-co-glycolic acid (PLGA) nanoparticle was conjugated to influenza A (H1N1)-conserved peptides and was intranasally administered as a vaccine. This vaccine was able to induce antigen-specific CD4+ and CD8+ T-cells and consequently induce protection in the lungs of pigs [146]. Therefore, the induction of cross-protective antibodies that can target several pathways involved in the infection would be highly beneficial for better protection against the influenza virus. Two different conserved influenza antigens (helix C and the ectodomain of matrix protein 2 (M2e)) were linked in a self-assembled nanoparticle to achieve this goal. They were able to induce neutralizing antibodies in mouse models [147].

### 9.4. Universal Influenza Vaccines

Although vaccination is the most efficient way to prevent influenza infection, antigenic shift and antigenic drift of influenza viruses allow them to escape antibody neutralization [148]. These variations in the influenza viruses are unpredictable, so influenza outbreak management is very challenging. To overcome the limitations of the current influenza vaccines, a universal influenza vaccine that provides long duration and broad protection would be highly desirable. An ideal universal influenza vaccine should be effective against all influenza viruses (A and B) HA and NA subtypes or antigenic mutations. To reach this goal, the vaccine should induce cross-protective antibodies, which might be achieved by targeting the conserved epitopes found in HA, NA, or M2 or internal proteins such as M1 and NP.

The stem or stalk region of the HA is highly conserved across different strains of influenza viruses and is considered a very promising target for developing a universal influenza vaccine [149]. Furthermore, some of the isolated antibodies from humans generated against the stalk region of the virus neutralize all influenza A virus subtypes, which can be used for developing a universal influenza vaccine [150]. For example, a sequential chimeric HA vaccine technique was developed to redirect the immune response from the head to the stalk domain (Table 3). Chimeric HAs are composed of stalk domains from group 1 or group 2 influenza viruses and head domains from subtypes of avian influenza virus. After completing a phase I clinical trial in 2020, this universal influenza vaccine candidate was found to be safe and evoke a broad, vigorous, long-lasting, and effective immune response directed against the conserved, immuno-subdominant stalk of the hemagglutinin [151].

Moreover, a computationally optimized broadly reactive Ags (COBRA) targeting H1 was developed as a universal influenza vaccine (Table 3). To evaluate the breadth of B cells, antibody-secreting cells elicited by a COBRA hemagglutinin (HA) (termed P1) were compared to antibody-secreting cells elicited by historical H1N1 vaccine strains. P1 HA-elicited monoclonal antibodies (mAbs) demonstrated an extensive range of HA identification, spanning from narrowly to widely reactive mAbs. A new vaccine, Multimeric-001, was also developed to defend against seasonal and pandemic influenza virus types, independent of mutations, by containing conserved linear epitopes from the HA, NP, and M1 proteins of influenza type A and type B strains (Table 3). This vaccine was evaluated in phase III clinical trial in 2020 to assess its protection and tolerability, as well as the humoral and cellular immune responses [154]. The vaccine was well tolerated, and no serious adverse effects were recorded. The humoral and cellular responses indicate that the vaccine has cross-immunity against influenza virus strains independent of mutations. Participants given Multimeric-001 dose prior to a seasonal vaccination had greater antibody response to match the H1N1 and H3N2 strains compared to seasonal vaccination alone. Moreover, there was an increase in CD4+ and CD8+ T cell responses to H1N1, H3N2, and influenza B relative to baseline in those who were vaccinated with Multimeric-001.

## 10. Characterization of Vaccine Products

Most of the vaccine production time (70%) is devoted to quality testing [155] to assure purity, potency, and vaccine safety. During the development of influenza vaccines, extensive characterizations are required to ensure that the manufacturing steps follow current good manufacturing practice (cGMP).

Although the production of vaccines differs, quality control tests must consider certain aspects of physicochemical parameters, such as purity, quantity, homogeneity, and stability.

### 10.1. Purity

The purity of the vaccine is a critical physicochemical criterion for determining the proportion of “active” vaccine antigens in the final vaccine product. Ultimately, the degree of pureness measured by the vaccine antigen will rely on the test’s specificity, additional methods for quantification and preparation of the sample of the product, and nonproduct impurities. Thus, it is critical to employ selective assays to measure putative nonproduct/process-related impurities (e.g., host cell proteins, host cell DNA and/or RNA, lipids, cell culture inducers, etc.) and product-related impurities (e.g., molecular weight variants such as aggregates and degradants) to delineate the existence of contaminants and impurities below a defined amount [156]. Moreover, IIV need to be purified by removing the inactivating agent using filtration, centrifugation, or evaporation methods. Splitting materials in split-virus vaccines such as Triton-100 need to be removed in the final product, which can be done by filtration or other separation methods such as hydrophobic interaction chromatography [157]. Table 4 summarizes several approaches that may be used, often in combination, to determine vaccine purity. For example, using orthogonal processes such as mass spectrometry, the molecular mass can be accurately measured, and the covalent change (i.e., glycosylation) identity and locations may also be detailed. Furthermore, since an impurity can exhibit the same mass/charge (*m/z*) ratio as the vaccine antigen during mass spectrometric analysis, using another orthogonal method such as light scattering (size-exclusion chromatography, multiple-angle laser light scattering) that measures mass based on the radius of gyration helps in separating the vaccine antigen from the unresolved impurity during mass spectrometric analysis.

### 10.2. Quantity

HA is the primary antigenic protein in the influenza virus, and the quantity of HA should be enough in the vaccine to induce the immune system. Thus, HA quantity must be 15 µg/mL for any type of influenza vaccine [157]. Quantification is carried out using conventional protein estimation approaches such as UV Abs 280 nm, bicinchoninic acid assay, Bradford assay, Lowry assay, detergent-based fluorescent detection, quantitative gel electrophoresis with fluorescent staining, amine-labeling “derivatization” (using fluorescent probes), and ELISA (enzyme linked immunosorbent assay). Although each approach has some possible advantages and disadvantages, it is critical to understand the method(s) of measuring in relation to the sample volume, pH environments, sensitivity, throughput, robustness, and precision of the assay [176]. Finally, assessing the quantity of the “working” vaccine antigen in the presence of product-related impurities is critical for developing correct dose guidelines.

### 10.3. Homogeneity

The size or molecular weight of the proteins in the vaccine antigen can be determined using many techniques: separation and scattering methods are the main two methods [177]. In the separation method, a force can be applied (e.g., mechanical, centrifugal, electrical) on the sample to separate molecules based on various characteristics such as size, charge, hydrophobicity, then the size, shape, or charge of the biomolecule can be determined depending on its movement. Separation methods include chromatography assays such as high-performance liquid chromatography, gel electrophoresis, and sedimentation equilibrium. In addition to light scattering methods, other spectroscopy methods such as mass spectroscopy and fluorescence spectroscopy are also frequently employed when characterizing samples. Moreover, microscopic methods such as transmission electron microscopy (TEM) and flow imaging microscopy have valuable applications in terms of protein characterization (Table 4). Field-flow fractionation is a technique that separates proteins/glycoproteins/particles depending on the orientation of the laminar flow of the molecules in a narrow channel [178], with smaller molecules flowing faster and larger molecules (that cannot disperse rapidly) moving slowly. The usage of mass spectrometry to assess the molecular mass or scale of proteins aids in protein recognition and quantifies protein purity, covalent alteration, and degradation products at both quantitative and qualitative stages [179]. Light scattering and electron microscopy (EM) are the two techniques often used for characterizing and determining the size of protein/glycoprotein-based vaccine antigens or the size distribution of virions in WIVs.

Moreover, the presence of protein aggregation in a vaccine product can affect the stability and efficacy of the vaccine [172]. These aggregates can be caused by removing the splitting agent (surfactant) in the manufacturing of split-virus influenza vaccines [180]. In addition to EM and light scattering, UV−Vis absorption spectroscopy and fluorescence emission spectroscopy are reliable techniques in identifying protein aggregations [89,172]. EM is a direct imaging tool for determining the size and shape of large proteins or a complex of proteins. However, owing to the high expense of the instrumentation and the specialized knowledge needed for such experiments, other techniques (such as light scattering) are often chosen as primary methods for size analysis. Molecular weight, distribution, diffusion coefficients and hydrodynamic radius can be measured through dynamic light scattering.

### 10.4. Stability

Understanding the function and stability of the protein/glycoprotein vaccine antigen is crucial to the vaccine’s general effectiveness in eliciting the required protective immune response. The instability of vaccines may be induced by different factors such as heat, light, radiation, environmental changes, or interactions with the container or other vaccine ingredients. Excessive heat or freezing might hasten the loss of a vaccine’s potency. It is thus necessary to treat vaccines with care, pay close attention to their handling to guarantee that they are of high quality, and provide maximum efficacy. This may be accomplished via the use of cold chains to keep vaccines in a restricted temperature environment (which is required for potency preservation) from the producer all the way down to the end consumers. Moreover, in split-virus vaccines, surfactant type, incubation time, virus concentration, surfactant concentration, and virus split ratio are other factors that could affect a vaccine’s stability. For example, aggregation may present after diluting or removal of the surfactant, so the remaining surfactant concentration should be monitored by optimizing the number of filtering steps to maintain vaccine stability and efficacy [180].

A combination of analytical methods can be used to study vaccine stability, such as fluorescence emission spectroscopy, UV−Vis absorption spectroscopy, and hemagglutinin inhibition assay (HI) test (Table 4). Because aggregation and/or degradation are typical characteristics that influence the stability for proteins/glycoproteins, utilizing these analysis methods helps identify the size or molecular weight of the aggregation to understand a vaccine’s short-term and long-term stability [181].

## 11. Effectiveness of Influenza Vaccines 

**Figure 6:**
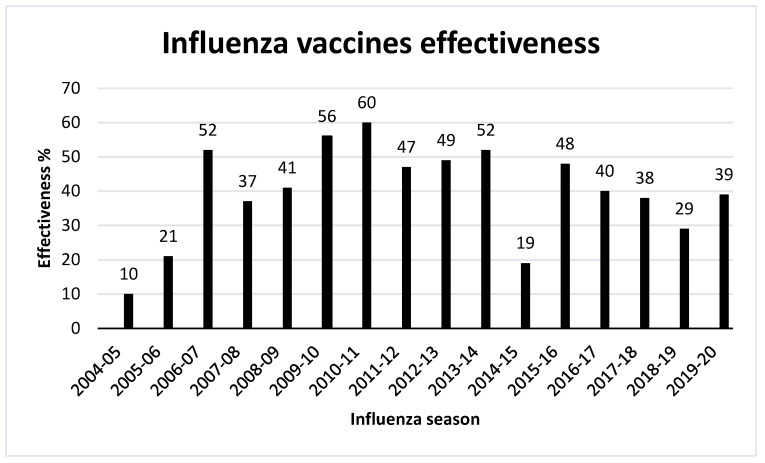
Fluctuation of seasonal influenza vaccines effectiveness from 2004–2020 in US, according to CDC [183].

Vaccine effectiveness has been one of the major issues in influenza vaccines and hence the effectiveness of the seasonal influenza vaccination is evaluated every year. Efficacy measurement of vaccines may be affected by some factors such as the population investigated, the research design, the outcome(s) assessed, and the flu vaccination season analyzed [182]. Typically, flu vaccination lowers the risk of influenza disease by between 40 to 60% in the general population during flu seasons when the majority of circulating influenza viruses are closely matched to those used in flu vaccines [183]. However, a poorly matched flu vaccine will offer little or no protection against disease caused by the commonly circulating viruses. Therefore, influenza vaccine effectiveness has varied from year to year, ranging from a low of 10% in 2004–2005 to a high of 60% in 2010–2011 seasons (Figure 6).

Many parameters contribute to inconsistency in vaccination responses including intrinsic viral characteristics as well as host factors [184]. A growing body of data suggests that host variables such as age, gender, pregnancy, and immunological history play significant roles in modifying the effectiveness of influenza vaccines. Thus, a better understanding of the host variables that influence influenza vaccine-induced immunity may help enhance effectiveness of existing seasonal and future universal influenza vaccines by optimizing or even customizing vaccine type, dosage, and adjuvant usage [185]. The influenza vaccine’s effectiveness is usually measured using the test-negative design (TND) since 2004, when health officials started tracking the effectiveness of influenza vaccines. TND is a modified case–control approach, it contrasts the vaccination status of individuals with influenza-like symptoms who come to healthcare settings. Although it minimizes selection bias associated with health-seeking activities, it has limitations in that it only includes people who seek care for respiratory illnesses. However, randomized controlled trials would be more appropriate to measure a vaccine’s effectiveness as they are less prone to selection and confounding biases [182].

To improve the effectiveness of influenza vaccines, different strategies can be used. For instance, increasing the vaccine’s HA antigen dosage to achieve greater haemagglutinin inhibition titers and immunogenicity [186]. It was shown in a recent meta-analysis that higher-dose IIV was more beneficial than standard-dose IIV in avoiding hospitalization from influenza, as well as a reduction in influenza-related mortality in individuals over the age of 65 [186]. Using intradermal vaccination to improve vaccine effectiveness is another option, showing haemagglutinin inhibition levels that are equal to or better than those obtained via intramuscular injection [187]. This was attributed to an increase in the number of antigen-presenting cells inside the dermis, which is one of the mechanisms responsible for enhanced immunogenicity of intradermal vaccination. The addition of adjuvants to vaccines is another method for increasing the efficacy of vaccines. The introduction of novel adjuvant trivalent inactivated vaccines has been demonstrated to enhance immune response in the elderly when compared to nonadjuvanted trivalent inactivated vaccines, and to decrease the risk of hospitalization due to influenza and pneumonia by half [80]. Another option would be using a non-egg-based system to avoid mutations and other changes such as post-translational modifications including glycosylation on viral proteins during egg adaptation of virus.

## 12. Conclusions

Due to extensive viral shift and drift, influenza can evade the host’s immune defenses, and hence, remains one of the significant, recurring global public healthcare challenges. Therefore, there is a need to update the influenza vaccines annually. Currently, three types of influenza vaccines are on the market: inactivated influenza vaccine (IIV), live attenuated influenza vaccine (LAIV), and recombinant hemagglutinin (HA) vaccine. Like most other vaccines, the influenza vaccine has a complex formulation that requires several key components such as a viral antigen, adjuvant, preservative, and stabilizer to maintain a stable and effective vaccine formulation. Further, the vaccine undergoes many quality tests throughout the processing steps and the final product to ensure that the vaccine is up to standard and safe to use. Nevertheless, more research is needed to overcome the limitations of current influenza vaccines, such as low efficacy, long production time, and the lack of cross-protection. To this end, new influenza vaccine candidates are in progress to improve the vaccine efficacy or to provide cross-protection against many different strains (i.e., a universal influenza vaccine) to overcome the need for an annual update for the vaccine.

## Figures and Tables

**Table 1:** Various licensed seasonal influenza vaccines available in the US (representing the northern hemisphere) and Australia (representing the southern hemisphere).

No	Tradename	Manufacturer	Age Group	Country	References
*Inactivated Influenza Vaccine, Split Virion, Egg-Based*					
1	Afluria Quadrivalent	Seqirus Pty. Ltd.	Six months and over	US	[72]
2	Fluarix Quadrivalent	GlaxoSmithKline Biologicals	Six months and over	US	[73]
3	FluLaval Quadrivalent	ID Biomedical Corporation of Quebec	Six months and over	US	[74]
4	Fluzone Highdose Quadrivalent	Sanofi Pasteur, Inc.	65 years and over	US	[75]
5	Fluzone Quadrivalent	Sanofi Pasteur, Inc.	Six months and over	US	[76]
6	FluQuadri	Sanofi-Aventis Australia	Six months and over	Australia	[77]
7	Vaxigrip Tetra	Sanofi-Aventis Australia	Six months and over	Australia	[78]
8	Fluarix Tetra	GlaxoSmithKline Biologicals	Six months and over	Australia	[74]
9	Afluria Quadrivalent	Seqirus Pty. Ltd.	Five years and over	Australia	[79]
10	Influvac Tetra	Mylan Health	Three years and over	Australia	[79]
*Inactivated Influenza Vaccine, Surface Antigen, Adjuvanted, Egg-Based*					
11	Fluad	Seqirus, Inc.	65 years and over	US	[80]
12	Fluad Quadrivalent	Seqirus, Inc.	65 years and over	US	[81]
13	Fluad Quad	Seqirus, Inc.	65 years and over	Australia	[80]
*Recombinant Vaccine*					
14	Flublok Quadrivalent	Sanofi Pasteur, Inc.	18 years and over	US	[82]
*Inactivated Subunit Influenza Vaccine, Cell Culture-Based*					
15	Flucelvax Quadrivalent	Seqirus, Inc.	Four years and over	US	[83]
*Live Attenuated Influenza Vaccine*					
16	FluMist Quadrivalent	MedImmune, LLC	Two years through 49 years	US	[84]

**Table 2:** Current VLP influenza vaccine candidates in preclinical and clinical trials.

VLP Vaccine Candidate	Type of Response	Cross-Protection	Clinical Phase	Type of Species	Sponsor	References
BV VLP-HA-NA-M1	Humoral and cellular	N/A	Preclinical	Mice	-	[132]
HBc VLP-M2e-HA2 (Tandiflu1)	Cellular	Yes	Preclinical	Mice	-	[133]
HBc VLP-M2e-NP	Humoral and cellular	Yes	Preclinical	Mice	-	[127]
Influenza VLP-HA (H1, H8, H13, H3, H4, H10)	Humoral	Yes	Preclinical	Mice	-	[134]
Recombinant A (H1N1) 2009 influenza VLP vaccine (HA)	Humoral	N/A	Phase II	Human	Novavax	[135]
HA and M2e5x	Humoral and cellular	N/A	Preclinical	Mice	-	[136]
Plant-based QVLP (HA)	Humoral and cellular	Yes	Phase I &II	Human	Medicago	[131,137]

**Table 3:** Universal influenza vaccine candidates in preclinical and clinical trials.

Vaccine Candidate	Vaccine Type	Manufacturer	Clinical Phase	Participants	Mechanism of Action
Chimeric HA proteins [151]	Hemagglutinin based	Glaxo-SmithKline	Phase I	66	Ag-specific cellular response Broadly cross-reactive Abs
Computationally optimized broadly reactive antigens (COBRA) [152]	Computationally optimized antigens	Sanofi-Pasteur	Preclinical	-	Elicitation of a unique broad cross-reactive and cross-neutralizing Ab against HA.
NP, M1 and HA peptides (Multimeric-001) [153]	Recombinant proteins	BiondVax Pharmaceuticals Ltd./NIAID	Phase III	12463	Cellular (B- and T-cell) immune response.

**Table 4:** Techniques in vaccine development and characterization.

Assay or Technique	Use	References
**Identity**		
Hemagglutinin inhibition assay (HI Test)	Selection of the candidate virus vaccine CVV and stability	[158,159,160]
Single radial immunodiffusion assay SRID	Vaccine potency	[161,162,163]
Enzyme-linked immunosorbent assay ELISA		
**Sedimentation**		
Velocity	Mass and size	[164]
Equilibrium	Mass, association	[164]
**Chromatography**		
Hydrophobic interaction	Purification	[165]
Reverse phase	Purification and stability	[166]
Ion exchange	Charge	[167]
Size exclusion	Size	[168]
Affinity	Specific interaction	[169]
**Light scattering**		
Dynamic light scattering DLS	Size measurements and aggregation study	[170,171]
Static light scattering		
UV-Visible absorbance spectroscopy	Agglomeration assessment and stability	[171,172,173]
Mass spectrometry	Protein quantification, vaccine potency	[34,174]
Fluorescence spectroscopy	Protein stability, agglomeration assessment	[89,172]
**Microscopy**		
Transmission electron microscopy TEM	Structure-guided information and aggregation study	[89,171,172]
Flow imaging microscopy	Size	[175]

## References

[1] Klimov A.I., Garten R., Russell C., Barr I.G., Besselaar T.G., Daniels R., Engelhardt O.G., Grohmann G., Itamura S., Kelso A. (2012). WHO recommendations for the viruses to be used in the 2012 Southern Hemisphere Influenza Vaccine: Epidemiology, antigenic and genetic characteristics of influenza A(H1N1)pdm09, A(H3N2) and B influenza viruses collected from February to September 2011. Vaccine.

[2] CDC Types of Influenza Viruses. https://www.cdc.gov/flu/about/viruses/types.htm.

[3] Asha K., Kumar B. (2019). Emerging Influenza D Virus Threat: What We Know So Far!. J. Clin. Med..

[4] Dawson W.K., Lazniewski M., Plewczynski D. (2017). RNA structure interactions and ribonucleoprotein processes of the influenza A virus. Brief. Funct. Genom..

[5] Saunders-Hastings P.R., Krewski D. (2016). Reviewing the History of Pandemic Influenza: Understanding Patterns of Emergence and Transmission. Pathogens.

[6] Seladi-Schulman J., Steel J., Lowen A.C. (2013). Spherical influenza viruses have a fitness advantage in embryonated eggs, while filament-producing strains are selected in vivo. J. Virol..

[7] Gaymard A., Le Briand N., Frobert E., Lina B., Escuret V. (2016). Functional balance between neuraminidase and haemagglutinin in influenza viruses. Clin. Microbiol. Infect..

[8] Harris A., Cardone G., Winkler D.C., Heymann J.B., Brecher M., White J.M., Steven A.C. (2006). Influenza virus pleiomorphy characterized by cryoelectron tomography. Proc. Natl. Acad. Sci. USA.

[9] Moulès V., Terrier O., Yver M., Riteau B., Moriscot C., Ferraris O., Julien T., Giudice E., Rolland J.-P., Erny A. (2011). Importance of viral genomic composition in modulating glycoprotein content on the surface of influenza virus particles. Virology.

[10] Tong S., Li Y., Rivailler P., Conrardy C., Castillo D.A., Chen L.M., Recuenco S., Ellison J.A., Davis C.T., York I.A. (2012). A distinct lineage of influenza A virus from bats. Proc. Natl. Acad. Sci. USA.

[11] De Vries R.D., Herfst S., Richard M. (2018). Avian Influenza A Virus Pandemic Preparedness and Vaccine Development. Vaccines.

[12] Asaduzzaman S.M., Ma J., van den Driessche P. (2015). The coexistence or replacement of two subtypes of influenza. Math. Biosci..

[13] Petric M., Comanor L., Petti C.A. (2006). Role of the Laboratory in Diagnosis of Influenza during Seasonal Epidemics and Potential Pandemics. J. Infect. Dis..

[14] Zebedee S.L., Lamb R.A. (1988). Influenza A virus M2 protein: Monoclonal antibody restriction of virus growth and detection of M2 in virions. J. Virol..

[15] Shaw M., Palese P., Knipe D.M., Howley P.M. (2007). Orthomyxoviridae: The viruses and their replication. Fields Virol.

[16] Bouvier N.M., Palese P. (2008). The biology of influenza viruses. Vaccine.

[17] Wolff T., Veit M. (2021). Influenza B, C and D Viruses (*Orthomyxoviridae*). Encyclopedia of Virology.

[18] Kuchipudi S.V., Nelli R.K., Gontu A., Satyakumar R., Surendran Nair M., Subbiah M. (2021). Sialic Acid Receptors: The Key to Solving the Enigma of Zoonotic Virus Spillover. Viruses.

[19] Samji T. (2009). Influenza A: Understanding the viral life cycle. Yale J. Biol. Med..

[20] Engelhardt O.G., Smith M., Fodor E. (2005). Association of the influenza A virus RNA-dependent RNA polymerase with cellular RNA polymerase II. J. Virol..

[21] Nayak D.P., Balogun R.A., Yamada H., Zhou Z.H., Barman S. (2009). Influenza virus morphogenesis and budding. Virus Res..

[22] Gamblin S.J., Skehel J.J. (2010). Influenza hemagglutinin and neuraminidase membrane glycoproteins. J. Biol. Chem..

[23] Hai R., Krammer F., Tan G.S., Pica N., Eggink D., Maamary J., Margine I., Albrecht R.A., Palese P. (2012). Influenza viruses expressing chimeric hemagglutinins: Globular head and stalk domains derived from different subtypes. J. Virol..

[24] Velkov T., Ong C., Baker M.A., Kim H., Li J., Nation R.L., Huang J.X., Cooper M.A., Rockman S. (2013). The antigenic architecture of the hemagglutinin of influenza H5N1 viruses. Mol. Immunol..

[25] Wohlbold T.J., Krammer F. (2014). In the shadow of hemagglutinin: A growing interest in influenza viral neuraminidase and its role as a vaccine antigen. Viruses.

[26] Von Itzstein M., Thomson R. (2009). Anti-influenza drugs: The development of sialidase inhibitors. Handb. Exp. Pharm..

[27] Byrd-Leotis L., Cummings R.D., Steinhauer D.A. (2017). The Interplay between the Host Receptor and Influenza Virus Hemagglutinin and Neuraminidase. Int. J. Mol. Sci..

[28] York I.A., Stevens J., Alymova I.V. (2019). Influenza virus N-linked glycosylation and innate immunity. Biosci. Rep..

[29] Vigerust D.J., Shepherd V.L. (2007). Virus glycosylation: Role in virulence and immune interactions. Trends Microbiol..

[30] Kim P., Jang Y.H., Kwon S.B., Lee C.M., Han G., Seong B.L. (2018). Glycosylation of Hemagglutinin and Neuraminidase of Influenza A Virus as Signature for Ecological Spillover and Adaptation among Influenza Reservoirs. Viruses.

[31] Job E.R., Deng Y.M., Barfod K.K., Tate M.D., Caldwell N., Reddiex S., Maurer-Stroh S., Brooks A.G., Reading P.C. (2013). Addition of glycosylation to influenza A virus hemagglutinin modulates antibody-mediated recognition of H1N1 2009 pandemic viruses. J. Immunol..

[32] Wanzeck K., Boyd K.L., McCullers J.A. (2011). Glycan shielding of the influenza virus hemagglutinin contributes to immunopathology in mice. Am. J. Respir. Crit. Care Med..

[33] Kobayashi Y., Suzuki Y. (2012). Evidence for N-glycan shielding of antigenic sites during evolution of human influenza A virus hemagglutinin. J. Virol..

[34] Cruz E., Cain J., Crossett B., Kayser V. (2018). Site-specific glycosylation profile of influenza A (H1N1) hemagglutinin through tandem mass spectrometry. Hum. Vaccines Immunother..

[35] Fiore A.E., Uyeki T.M., Broder K., Finelli L., Euler G.L., Singleton J.A., Iskander J.K., Wortley P.M., Shay D.K., Bresee J.S. (2010). Prevention and control of influenza with vaccines: Recommendations of the Advisory Committee on Immunization Practices (ACIP), 2010. MMWR Recomm. Rep..

[36] Barr I.G., McCauley J., Cox N., Daniels R., Engelhardt O.G., Fukuda K., Grohmann G., Hay A., Kelso A., Klimov A. (2010). Epidemiological, antigenic and genetic characteristics of seasonal influenza A(H1N1), A(H3N2) and B influenza viruses: Basis for the WHO recommendation on the composition of influenza vaccines for use in the 2009–2010 Northern Hemisphere season. Vaccine.

[37] Chang D., Zaia J. (2019). Why Glycosylation Matters in Building a Better Flu Vaccine. Mol. Cell Proteom..

[38] Saito T., Taylor G., Webster R.G. (1995). Steps in maturation of influenza A virus neuraminidase. J. Virol..

[39] Wang N., Glidden E.J., Murphy S.R., Pearse B.R., Hebert D.N. (2008). The cotranslational maturation program for the type II membrane glycoprotein influenza neuraminidase. J. Biol. Chem..

[40] Bao D., Xue R., Zhang M., Lu C., Ma T., Ren C., Zhang T., Yang J., Teng Q., Li X. (2021). N-Linked Glycosylation Plays an Important Role in Budding of Neuraminidase Protein and Virulence of Influenza Viruses. J. Virol..

[41] Iuliano A.D., Roguski K.M., Chang H.H., Muscatello D.J., Palekar R., Tempia S., Cohen C., Gran J.M., Schanzer D., Cowling B.J. (2018). Estimates of global seasonal influenza-associated respiratory mortality: A modelling study. Lancet.

[42] WHO Influenza (Seasonal). https://www.who.int/news-room/fact-sheets/detail/influenza-(seasonal).

[43] Fleming D.M., Zambon M., Bartelds A.I.M., De Jong J.C. (1999). The duration and magnitude of influenza epidemics: A study of surveillance data from sentinel general practices in England, Wales and the Netherlands. Eur. J. Epidemiol..

[44] Boni M.F. (2008). Vaccination and antigenic drift in influenza. Vaccine.

[45] McCaughey C. (2010). Influenza: A virus of our times. Ulst. Med. J..

[46] Potter C.W. (2001). A history of influenza. J. Appl. Microbiol..

[47] Nguyen-Van-Tam J.S., Hampson A.W. (2003). The epidemiology and clinical impact of pandemic influenza. Vaccine.

[48] Scholtissek C., Rohde W., Von Hoyningen V., Rott R. (1978). On the origin of the human influenza virus subtypes H2N2 and H3N2. Virology.

[49] De Wit E., Fouchier R.A. (2008). Emerging influenza. J. Clin. Virol..

[50] Jester B., Uyeki T.M., Jernigan D.B., Tumpey T.M. (2019). Historical and clinical aspects of the 1918 H1N1 pandemic in the United States. Virology.

[51] Johnson N.P., Mueller J. (2002). Updating the accounts: Global mortality of the 1918–1920 “Spanish” influenza pandemic. Bull. Hist. Med..

[52] Kayser V., Ramzan I. (2021). Vaccines and Vaccination: History and Emerging Issues. Hum. Vaccines Immunother..

[53] Spreeuwenberg P., Kroneman M., Paget J. (2018). Reassessing the Global Mortality Burden of the 1918 Influenza Pandemic. Am. J. Epidemiol..

[54] Taubenberger J.K., Kash J.C., Morens D.M. (2019). The 1918 influenza pandemic: 100 years of questions answered and unanswered. Sci. Transl. Med..

[55] Viboud C., Simonsen L., Fuentes R., Flores J., Miller M.A., Chowell G. (2016). Global Mortality Impact of the 1957-1959 Influenza Pandemic. J. Infect. Dis..

[56] Oxford J.S. (2000). Influenza A pandemics of the 20th century with special reference to 1918: Virology, pathology and epidemiology. Rev. Med. Virol..

[57] CDC 1968 Pandemic (H3N2 Virus). https://www.cdc.gov/flu/pandemic-resources/1968-pandemic.html.

[58] Mena I., Nelson M.I., Quezada-Monroy F., Dutta J., Cortes-Fernández R., Lara-Puente J.H., Castro-Peralta F., Cunha L.F., Trovão N.S., Lozano-Dubernard B. (2016). Origins of the 2009 H1N1 influenza pandemic in swine in Mexico. eLife.

[59] WHO What Is the Pandemic (H1N1) 2009 Virus?. https://www.who.int/csr/disease/swineflu/frequently_asked_questions/about_disease/en/.

[60] Taubenberger J.K., Morens D.M. (2010). Influenza: The once and future pandemic. Public Health Rep..

[61] Paules C., Subbarao K. (2017). Influenza. Lancet.

[62] Peiris J.S.M., Yu W.C., Leung C.W., Cheung C.Y., Ng W.F., Nicholls J.M., Ng T.K., Chan K.H., Lai S.T., Lim W.L. (2004). Re-emergence of fatal human influenza A subtype H5N1 disease. Lancet.

[63] Chowdhury S., Hossain M.E., Ghosh P.K., Ghosh S., Hossain M.B., Beard C., Rahman M., Rahman M.Z. (2019). The Pattern of Highly Pathogenic Avian Influenza H5N1 Outbreaks in South Asia. Trop. Med. Infect. Dis..

[64] WHO Influenza (Avian and Other Zoonotic). https://www.who.int/news-room/fact-sheets/detail/influenza-(avian-and-other-zoonotic).

[65] CDC Highly Pathogenic Asian Avian Influenza A(H5N1) in People. https://www.cdc.gov/flu/avianflu/h5n1-people.htm.

[66] WHO Avian Influenza Weekly Update Number 716. https://www.who.int/docs/default-source/wpro---documents/emergency/surveillance/avian-influenza/ai-20191122.pdf?sfvrsn=30d65594_42.

[67] Young B., Sadarangani S., Jiang L., Wilder-Smith A., Chen M.I. (2018). Duration of Influenza Vaccine Effectiveness: A Systematic Review, Meta-analysis, and Meta-regression of Test-Negative Design Case-Control Studies. J. Infect. Dis..

[68] Weir J.P., Gruber M.F. (2016). An overview of the regulation of influenza vaccines in the United States. Influenza Other Respir. Viruses.

[69] Gutierrez A.F., El Sahly H. (2015). Recombinant hemagglutinin protein vaccine: A new option in immunization against influenza. Future Virol..

[70] Yamayoshi S., Kawaoka Y. (2019). Current and future influenza vaccines. Nat. Med..

[71] Xie H., Wan X.F., Ye Z.P., Plant E.P., Zhao Y.Q., Xu Y.F., Li X., Finch C., Zhao N., Kawano T. (2015). H3N2 Mismatch of 2014-15 Northern Hemisphere Influenza Vaccines and Head-to-head Comparison between Human and Ferret Antisera derived Antigenic Maps. Sci. Rep..

[72] Statler V.A., Albano F.R., Airey J., Sawlwin D.C., Graves Jones A., Matassa V., Heijnen E., Edelman J., Marshall G.S. (2019). Immunogenicity and safety of a quadrivalent inactivated influenza vaccine in children 6–59 months of age: A phase 3, randomized, noninferiority study. Vaccine.

[73] McKeage K. (2013). Inactivated quadrivalent split-virus seasonal influenza vaccine (Fluarix^®^ quadrivalent): A review of its use in the prevention of disease caused by influenza A and B. Drugs.

[74] Bekkat-Berkani R., Ray R., Jain V.K., Chandrasekaran V., Innis B.L. (2016). Evidence update: GlaxoSmithKline’s inactivated quadrivalent influenza vaccines. Expert Rev. Vaccines.

[75] Robertson C.A., DiazGranados C.A., Decker M.D., Chit A., Mercer M., Greenberg D.P. (2016). Fluzone^®^ High-Dose Influenza Vaccine. Expert Rev. Vaccines.

[76] Suryadevara M., Domachowske J.B. (2014). Quadrivalent influenza vaccine in the United States. Hum. Vaccines Immunother..

[77] Sullivan S.G., Arriola C.S., Bocacao J., Burgos P., Bustos P., Carville K.S., Cheng A.C., Chilver M.B., Cohen C., Deng Y.-M. (2019). Heterogeneity in influenza seasonality and vaccine effectiveness in Australia, Chile, New Zealand and South Africa: Early estimates of the 2019 influenza season. Eurosurveillance.

[78] Montomoli E., Torelli A., Manini I., Gianchecchi E. (2018). Immunogenicity and Safety of the New Inactivated Quadrivalent Influenza Vaccine Vaxigrip Tetra: Preliminary Results in Children ≥6 Months and Older Adults. Vaccines.

[79] Zhao L., Young K., Gemmill I. (2019). Summary of the NACI Seasonal Influenza Vaccine Statement for 2019-2020. Can. Commun. Dis. Rep..

[80] Tsai T.F. (2013). Fluad^®^-MF59^®^-Adjuvanted Influenza Vaccine in Older Adults. Infect. Chemother..

[81] Essink B., Fierro C., Rosen J., Figueroa A.L., Zhang B., Verhoeven C., Edelman J., Smolenov I. (2020). Immunogenicity and safety of MF59-adjuvanted quadrivalent influenza vaccine versus standard and alternate B strain MF59-adjuvanted trivalent influenza vaccines in older adults. Vaccine.

[82] Dunkle L., Izikson R., Post P., Cox M. (2015). Introducing Modern Recombinant Technology to the Realm of Seasonal Influenza Vaccine: Flublok^®^ For Prevention of Influenza in Adults. EC Microbiol..

[83] Lamb Y.N. (2019). Cell-Based Quadrivalent Inactivated Influenza Virus Vaccine (Flucelvax^®^ Tetra/Flucelvax Quadrivalent^®^): A Review in the Prevention of Influenza. Drugs.

[84] Baxter R., Eaton A., Hansen J., Aukes L., Caspard H., Ambrose C.S. (2017). Safety of quadrivalent live attenuated influenza vaccine in subjects aged 2–49years. Vaccine.

[85] Hoft D.F., Lottenbach K.R., Blazevic A., Turan A., Blevins T.P., Pacatte T.P., Yu Y., Mitchell M.C., Hoft S.G., Belshe R.B. (2017). Comparisons of the Humoral and Cellular Immune Responses Induced by Live Attenuated Influenza Vaccine and Inactivated Influenza Vaccine in Adults. Clin. Vaccine Immunol..

[86] Al-Mazrou A., Scheifele D.W., Soong T., Bjornson G. (1991). Comparison of adverse reactions to whole-virion and split-virion influenza vaccines in hospital personnel. CMAJ.

[87] Soema P.C., Kompier R., Amorij J.P., Kersten G.F. (2015). Current and next generation influenza vaccines: Formulation and production strategies. Eur. J. Pharm. Biopharm..

[88] Sabbaghi A., Miri S.M., Keshavarz M., Zargar M., Ghaemi A. (2019). Inactivation methods for whole influenza vaccine production. Rev. Med. Virol..

[89] Lee K.K., Sahin Y.Z., Neeleman R., Trout B.L., Kayser V. (2016). Quantitative determination of the surfactant-induced split ratio of influenza virus by fluorescence spectroscopy. Hum. Vaccines Immunother..

[90] Squarcione S., Sgricia S., Biasio L.R., Perinetti E. (2003). Comparison of the reactogenicity and immunogenicity of a split and a subunit-adjuvanted influenza vaccine in elderly subjects. Vaccine.

[91] Khalaj-Hedayati A., Chua C.L.L., Smooker P., Lee K.W. (2019). Nanoparticles in influenza subunit vaccine development: Immunogenicity enhancement. Influenza Other Respir. Viruses.

[92] Maassab H.F. (1967). Adaptation and growth characteristics of influenza virus at 25 degrees C. Nature.

[93] Beyer W.E.P., Palache A.M., de Jong J.C., Osterhaus A.D.M.E. (2002). Cold-adapted live influenza vaccine versus inactivated vaccine: Systemic vaccine reactions, local and systemic antibody response, and vaccine efficacy: A meta-analysis. Vaccine.

[94] Suzuki T., Kawaguchi A., Ainai A., Tamura S.-i., Ito R., Multihartina P., Setiawaty V., Pangesti K.N.A., Odagiri T., Tashiro M. (2015). Relationship of the quaternary structure of human secretory IgA to neutralization of influenza virus. Proc. Natl. Acad. Sci. USA.

[95] King J.C., Treanor J., Fast P.E., Wolff M., Yan L., Iacuzio D., Readmond B., O’Brien D., Mallon K., Highsmith W.E. (2000). Comparison of the safety, vaccine virus shedding, and immunogenicity of influenza virus vaccine, trivalent, types A and B, live cold-adapted, administered to human immunodeficiency virus (HIV)-infected and non-HIV-infected adults. J. Infect. Dis..

[96] Belshe R.B., Mendelman P.M., Treanor J., King J., Gruber W.C., Piedra P., Bernstein D.I., Hayden F.G., Kotloff K., Zangwill K. (1998). The efficacy of live attenuated, cold-adapted, trivalent, intranasal influenzavirus vaccine in children. N. Engl. J. Med..

[97] Keitel W.A., Couch R.B., Cate T.R., Maassab H.F. (1994). Variability in infectivity of cold-adapted recombinant influenza virus vaccines in humans. J. Infect. Dis..

[98] Cox M.M., Hollister J.R. (2009). FluBlok, a next generation influenza vaccine manufactured in insect cells. Biologicals.

[99] Geisler C., Jarvis D.L. (2018). Adventitious viruses in insect cell lines used for recombinant protein expression. Protein Expr. Purif..

[100] Grohskopf L.A., Sokolow L.Z., Broder K.R., Walter E.B., Fry A.M., Jernigan D.B. (2018). Prevention and Control of Seasonal Influenza with Vaccines: Recommendations of the Advisory Committee on Immunization Practices-United States, 2018–2019 Influenza Season. MMWR Recomm Rep..

[101] Cox M.M., Patriarca P.A., Treanor J. (2008). FluBlok, a recombinant hemagglutinin influenza vaccine. Influenza Other Respir. Viruses.

[102] Treanor J.J., Wilkinson B.E., Masseoud F., Hu-Primmer J., Battaglia R., O’Brien D., Wolff M., Rabinovich G., Blackwelder W., Katz J.M. (2001). Safety and immunogenicity of a recombinant hemagglutinin vaccine for H5 influenza in humans. Vaccine.

[103] Wong S.S., Webby R.J. (2013). Traditional and new influenza vaccines. Clin. Microbiol. Rev..

[104] DeMarcus L., Shoubaki L., Federinko S. (2019). Comparing influenza vaccine effectiveness between cell-derived and egg-derived vaccines, 2017–2018 influenza season. Vaccine.

[105] Milian E., Kamen A.A. (2015). Current and emerging cell culture manufacturing technologies for influenza vaccines. Biomed Res. Int..

[106] Rajao D.S., Perez D.R. (2018). Universal Vaccines and Vaccine Platforms to Protect against Influenza Viruses in Humans and Agriculture. Front. Microbiol..

[107] CDC Cell-Based Flu Vaccines. https://www.cdc.gov/flu/protect/vaccine/cell-based.htm.

[108] Couch R.B. (2008). Seasonal inactivated influenza virus vaccines. Vaccine.

[109] Tate M.D., Job E.R., Deng Y.M., Gunalan V., Maurer-Stroh S., Reading P.C. (2014). Playing hide and seek: How glycosylation of the influenza virus hemagglutinin can modulate the immune response to infection. Viruses.

[110] Krause J.C., Crowe J.E. (2014). Committing the Oldest Sins in the Newest Kind of Ways-Antibodies Targeting the Influenza Virus Type A Hemagglutinin Globular Head. Microbiol. Spectr..

[111] Ping J., Lopes T.J.S., Nidom C.A., Ghedin E., Macken C.A., Fitch A., Imai M., Maher E.A., Neumann G., Kawaoka Y. (2015). Development of high-yield influenza A virus vaccine viruses. Nat. Commun..

[112] Stohr K., Bucher D., Colgate T., Wood J. (2012). Influenza virus surveillance, vaccine strain selection, and manufacture. Methods Mol. Biol..

[113] Skowronski D.M., Janjua N.Z., De Serres G., Sabaiduc S., Eshaghi A., Dickinson J.A., Fonseca K., Winter A.L., Gubbay J.B., Krajden M. (2014). Low 2012-13 influenza vaccine effectiveness associated with mutation in the egg-adapted H3N2 vaccine strain not antigenic drift in circulating viruses. PLoS ONE.

[114] Mochalova L., Gambaryan A., Romanova J., Tuzikov A., Chinarev A., Katinger D., Katinger H., Egorov A., Bovin N. (2003). Receptor-binding properties of modern human influenza viruses primarily isolated in Vero and MDCK cells and chicken embryonated eggs. Virology.

[115] Zost S.J., Parkhouse K., Gumina M.E., Kim K., Diaz Perez S., Wilson P.C., Treanor J.J., Sant A.J., Cobey S., Hensley S.E. (2017). Contemporary H3N2 influenza viruses have a glycosylation site that alters binding of antibodies elicited by egg-adapted vaccine strains. Proc. Natl. Acad. Sci. USA.

[116] Hegde N.R. (2015). Cell culture-based influenza vaccines: A necessary and indispensable investment for the future. Hum. Vaccines Immunother..

[117] An Y., Rininger J.A., Jarvis D.L., Jing X., Ye Z., Aumiller J.J., Eichelberger M., Cipollo J.F. (2013). Comparative glycomics analysis of influenza Hemagglutinin (H5N1) produced in vaccine relevant cell platforms. J. Proteome Res..

[118] Hutter J., Rodig J.V., Hoper D., Seeberger P.H., Reichl U., Rapp E., Lepenies B. (2013). Toward animal cell culture-based influenza vaccine design: Viral hemagglutinin N-glycosylation markedly impacts immunogenicity. J. Immunol..

[119] Paules C.I., Sullivan S.G., Subbarao K., Fauci A.S. (2018). Chasing Seasonal Influenza—The Need for a Universal Influenza Vaccine. N. Engl. J. Med..

[120] Lin Y., Wharton S.A., Whittaker L., Dai M., Ermetal B., Lo J., Pontoriero A., Baumeister E., Daniels R.S., McCauley J.W. (2017). The characteristics and antigenic properties of recently emerged subclade 3C.3a and 3C.2a human influenza A(H3N2) viruses passaged in MDCK cells. Influenza Other Respir. Viruses.

[121] Tregoning J.S., Russell R.F., Kinnear E. (2018). Adjuvanted influenza vaccines. Hum. Vaccines Immunother..

[122] Nakayama T. (2011). [Influenza vaccine and adjuvant]. Yakugaku Zasshi.

[123] Pelliccia M., Andreozzi P., Paulose J., D’Alicarnasso M., Cagno V., Donalisio M., Civra A., Broeckel R.M., Haese N., Jacob Silva P. (2016). Additives for vaccine storage to improve thermal stability of adenoviruses from hours to months. Nat. Commun..

[124] Chung E.H. (2014). Vaccine allergies. Clin. Exp. Vaccine Res..

[125] Grgacic E.V., Anderson D.A. (2006). Virus-like particles: Passport to immune recognition. Methods.

[126] Zhao Q., Li S., Yu H., Xia N., Modis Y. (2013). Virus-like particle-based human vaccines: Quality assessment based on structural and functional properties. Trends Biotechnol..

[127] Gao X., Wang W., Li Y., Zhang S., Duan Y., Xing L., Zhao Z., Zhang P., Li Z., Li R. (2013). Enhanced Influenza VLP vaccines comprising matrix-2 ectodomain and nucleoprotein epitopes protects mice from lethal challenge. Antivir. Res..

[128] Giurgea L.T., Morens D.M., Taubenberger J.K., Memoli M.J. (2020). Influenza Neuraminidase: A Neglected Protein and Its Potential for a Better Influenza Vaccine. Vaccines.

[129] Kumar A., Meldgaard T.S., Bertholet S. (2018). Novel Platforms for the Development of a Universal Influenza Vaccine. Front. Immunol..

[130] Low J.G., Lee L.S., Ooi E.E., Ethirajulu K., Yeo P., Matter A., Connolly J.E., Skibinski D.A., Saudan P., Bachmann M. (2014). Safety and immunogenicity of a virus-like particle pandemic influenza A (H1N1) 2009 vaccine: Results from a double-blinded, randomized Phase I clinical trial in healthy Asian volunteers. Vaccine.

[131] Pillet S., Couillard J., Trépanier S., Poulin J.-F., Yassine-Diab B., Guy B., Ward B.J., Landry N. (2019). Immunogenicity and safety of a quadrivalent plant-derived virus like particle influenza vaccine candidate—Two randomized Phase II clinical trials in 18 to 49 and ≥50 years old adults. PLoS ONE.

[132] Ren Z., Zhao Y., Liu J., Ji X., Meng L., Wang T., Sun W., Zhang K., Sang X., Yu Z. (2018). Intramuscular and intranasal immunization with an H7N9 influenza virus-like particle vaccine protects mice against lethal influenza virus challenge. Int. Immunopharmacol..

[133] Ramirez A., Morris S., Maucourant S., D’Ascanio I., Crescente V., Lu I.N., Farinelle S., Muller C.P., Whelan M., Rosenberg W. (2018). A virus-like particle vaccine candidate for influenza A virus based on multiple conserved antigens presented on hepatitis B tandem core particles. Vaccine.

[134] Luo Y., Mohan T., Zhu W., Wang C., Deng L., Wang B.Z. (2018). Sequential Immunizations with heterosubtypic virus-like particles elicit cross protection against divergent influenza A viruses in mice. Sci. Rep..

[135] López-Macías C., Ferat-Osorio E., Tenorio-Calvo A., Isibasi A., Talavera J., Arteaga-Ruiz O., Arriaga-Pizano L., Hickman S.P., Allende M., Lenhard K. (2011). Safety and immunogenicity of a virus-like particle pandemic influenza A (H1N1) 2009 vaccine in a blinded, randomized, placebo-controlled trial of adults in Mexico. Vaccine.

[136] Kang H.-J., Chu K.-B., Lee D.-H., Lee S.-H., Park B.R., Kim M.-C., Kang S.-M., Quan F.-S. (2019). Influenza M2 virus-like particle vaccination enhances protection in combination with avian influenza HA VLPs. PLoS ONE.

[137] Pillet S., Aubin É., Trépanier S., Bussière D., Dargis M., Poulin J.-F., Yassine-Diab B., Ward B.J., Landry N. (2016). A plant-derived quadrivalent virus like particle influenza vaccine induces cross-reactive antibody and T cell response in healthy adults. Clin. Immunol..

[138] Banchereau J., Steinman R.M. (1998). Dendritic cells and the control of immunity. Nature.

[139] Fonteneau J.F., Gilliet M., Larsson M., Dasilva I., Munz C., Liu Y.J., Bhardwaj N. (2003). Activation of influenza virus-specific CD4+ and CD8+ T cells: A new role for plasmacytoid dendritic cells in adaptive immunity. Blood.

[140] Abdel-Motal U.M., Guay H.M., Wigglesworth K., Welsh R.M., Galili U. (2007). Immunogenicity of influenza virus vaccine is increased by anti-gal-mediated targeting to antigen-presenting cells. J. Virol..

[141] Grodeland G., Mjaaland S., Tunheim G., Fredriksen A.B., Bogen B. (2013). The specificity of targeted vaccines for APC surface molecules influences the immune response phenotype. PLoS ONE.

[142] Muszkat M., Greenbaum E., Ben-Yehuda A., Oster M., Yeu’l E., Heimann S., Levy R., Friedman G., Zakay-Rones Z. (2003). Local and systemic immune response in nursing-home elderly following intranasal or intramuscular immunization with inactivated influenza vaccine. Vaccine.

[143] Su F., Patel G.B., Hu S., Chen W. (2016). Induction of mucosal immunity through systemic immunization: Phantom or reality?. Hum. Vaccines Immunother..

[144] Al-Halifa S., Gauthier L., Arpin D., Bourgault S., Archambault D. (2019). Nanoparticle-Based Vaccines against Respiratory Viruses. Front. Immunol..

[145] Rioux G., Mathieu C., Russell A., Bolduc M., Laliberte-Gagne M.E., Savard P., Leclerc D. (2014). PapMV nanoparticles improve mucosal immune responses to the trivalent inactivated flu vaccine. J. Nanobiotechnol..

[146] Hiremath J., Kang K.I., Xia M., Elaish M., Binjawadagi B., Ouyang K., Dhakal S., Arcos J., Torrelles J.B., Jiang X. (2016). Entrapment of H1N1 Influenza Virus Derived Conserved Peptides in PLGA Nanoparticles Enhances T Cell Response and Vaccine Efficacy in Pigs. PLoS ONE.

[147] Karch C.P., Li J., Kulangara C., Paulillo S.M., Raman S.K., Emadi S., Tan A., Helal Z.H., Fan Q., Khan M.I. (2017). Vaccination with self-adjuvanted protein nanoparticles provides protection against lethal influenza challenge. Nanomedicine.

[148] Kim H., Webster R.G., Webby R.J. (2018). Influenza Virus: Dealing with a Drifting and Shifting Pathogen. Viral Immunol..

[149] Ohmit S.E., Petrie J.G., Cross R.T., Johnson E., Monto A.S. (2011). Influenza hemagglutination-inhibition antibody titer as a correlate of vaccine-induced protection. J. Infect. Dis..

[150] Corti D., Voss J., Gamblin S.J., Codoni G., Macagno A., Jarrossay D., Vachieri S.G., Pinna D., Minola A., Vanzetta F. (2011). A neutralizing antibody selected from plasma cells that binds to group 1 and group 2 influenza A hemagglutinins. Science.

[151] Nachbagauer R., Feser J., Naficy A., Bernstein D.I., Guptill J., Walter E.B., Berlanda-Scorza F., Stadlbauer D., Wilson P.C., Aydillo T. (2021). A chimeric hemagglutinin-based universal influenza virus vaccine approach induces broad and long-lasting immunity in a randomized, placebo-controlled phase I trial. Nat. Med..

[152] Sautto G.A., Kirchenbaum G.A., Abreu R.B., Ecker J.W., Pierce S.R., Kleanthous H., Ross T.M. (2020). A Computationally Optimized Broadly Reactive Antigen Subtype-Specific Influenza Vaccine Strategy Elicits Unique Potent Broadly Neutralizing Antibodies against Hemagglutinin. J. Immunol..

[153] Van Doorn E., Liu H., Ben-Yedidia T., Hassin S., Visontai I., Norley S., Frijlink H.W., Hak E. (2017). Evaluating the immunogenicity and safety of a BiondVax-developed universal influenza vaccine (Multimeric-001) either as a standalone vaccine or as a primer to H5N1 influenza vaccine: Phase IIb study protocol. Medicine.

[154] Atsmon J., Kate-Ilovitz E., Shaikevich D., Singer Y., Volokhov I., Haim K.Y., Ben-Yedidia T. (2012). Safety and immunogenicity of multimeric-001—A novel universal influenza vaccine. J. Clin. Immunol..

[155] Preiss S., Garçon N., Cunningham A.L., Strugnell R., Friedland L.R. (2016). Vaccine provision: Delivering sustained & widespread use. Vaccine.

[156] Dey A.K., Malyala P., Singh M. (2014). Physicochemical and functional characterization of vaccine antigens and adjuvants. Expert Rev. Vaccines.

[157] Kon T.C., Onu A., Berbecila L., Lupulescu E., Ghiorgisor A., Kersten G.F., Cui Y.-Q., Amorij J.-P., Van der Pol L. (2016). Influenza Vaccine Manufacturing: Effect of Inactivation, Splitting and Site of Manufacturing. Comparison of Influenza Vaccine Production Processes. PLoS ONE.

[158] Pedersen J.C. (2014). Hemagglutination-inhibition assay for influenza virus subtype identification and the detection and quantitation of serum antibodies to influenza virus. Methods Mol. Biol..

[159] Kaufmann L., Syedbasha M., Vogt D., Hollenstein Y., Hartmann J., Linnik J.E., Egli A. (2017). An Optimized Hemagglutination Inhibition (HI) Assay to Quantify Influenza-specific Antibody Titers. J. Vis. Exp..

[160] Defang G.N., Martin N.J., Burgess T.H., Millar E.V., Pecenka L.A., Danko J.R., Arnold J.C., Kochel T.J., Luke T.C. (2012). Comparative Analysis of Hemagglutination Inhibition Titers Generated Using Temporally Matched Serum and Plasma Samples. PLoS ONE.

[161] Wood J.M., Weir J.P. (2018). Standardisation of inactivated influenza vaccines-Learning from history. Influenza Other Respir. Viruses.

[162] Minor P.D. (2015). Assaying the Potency of Influenza Vaccines. Vaccines.

[163] Engelhardt O.G., Edge C., Dunleavy U., Guilfoyle K., Harvey R., Major D., Newman R., Penn R., Skeldon S., Storey C. (2018). Comparison of single radial immunodiffusion, SDS-PAGE and HPLC potency assays for inactivated influenza vaccines shows differences in ability to predict immunogenicity of haemagglutinin antigen. Vaccine.

[164] Cole J.L., Lary J.W., P Moody T., Laue T.M. (2008). Analytical ultracentrifugation: Sedimentation velocity and sedimentation equilibrium. Methods Cell Biol..

[165] Weigel T., Soliman R., Wolff M.W., Reichl U. (2019). Hydrophobic-interaction chromatography for purification of influenza A and B virus. J. Chromatogr. B Anal. Technol. Biomed. Life Sci..

[166] Shytuhina A., Pristatsky P., He J., Casimiro D.R., Schwartz R.M., Hoang V.M., Ha S. (2014). Development and application of a reversed-phase high-performance liquid chromatographic method for quantitation and characterization of a Chikungunya virus-like particle vaccine. J. Chromatogr. A.

[167] Rustandi R.R., Wang F., Lancaster C., Kristopeit A., Thiriot D.S., Heinrichs J.H. (2016). Ion-Exchange Chromatography to Analyze Components of a Clostridium difficile Vaccine. Methods Mol. Biol..

[168] Lancaster C., Rustandi R.R., Pannizzo P., Ha S. (2016). A Size-Exclusion Chromatography Method for Analysis of Clostridium difficile Vaccine Toxins. Methods Mol. Biol..

[169] Zhao M., Vandersluis M., Stout J., Haupts U., Sanders M., Jacquemart R. (2019). Affinity chromatography for vaccines manufacturing: Finally ready for prime time?. Vaccine.

[170] Vajda J., Weber D., Brekel D., Hundt B., Müller E. (2016). Size distribution analysis of influenza virus particles using size exclusion chromatography. J. Chromatogr. A.

[171] Tay T., Agius C., Hamilton R., Bodle J., Rockman S. (2015). Investigation into alternative testing methodologies for characterization of influenza virus vaccine. Hum. Vaccines Immunother..

[172] Sahin Z., Neeleman R., Haines J., Kayser V. (2019). Preparation-free method can enable rapid surfactant screening during industrial processing of influenza vaccines. Vaccine.

[173] Sahin Z., Akkoc S., Neeleman R., Haines J., Kayser V. (2017). Nile Red fluorescence spectrum decomposition enables rapid screening of large protein aggregates in complex biopharmaceutical formulations like influenza vaccines. Vaccine.

[174] Downard K.M., Morrissey B., Schwahn A.B. (2009). Mass spectrometry analysis of the influenza virus. Mass Spectrom. Rev..

[175] Frahm G.E., Pochopsky A.W.T., Clarke T.M., Johnston M.J.W. (2016). Evaluation of Microflow Digital Imaging Particle Analysis for Sub-Visible Particles Formulated with an Opaque Vaccine Adjuvant. PLoS ONE.

[176] Noble J.E., Bailey M.J. (2009). Quantitation of protein. Methods Enzym..

[177] Rhodes D.G., Bossio R.E., Laue T.M. (2009). Determination of size, molecular weight, and presence of subunits. Methods Enzym..

[178] Liu J., Andya J.D., Shire S.J. (2006). A critical review of analytical ultracentrifugation and field flow fractionation methods for measuring protein aggregation. AAPS J..

[179] Baldwin M.A., Medzihradszky K.F., Lock C.M., Fisher B., Settineri T.A., Burlingame A.L. (2001). Matrix-assisted laser desorption/ionization coupled with quadrupole/orthogonal acceleration time-of-flight mass spectrometry for protein discovery, identification, and structural analysis. Anal. Chem..

[180] Tarasov M., Shanko A., Kordyukova L., Katlinski A. (2020). Characterization of Inactivated Influenza Vaccines Used in the Russian National Immunization Program. Vaccines.

[181] Filipe V., Hawe A., Carpenter J.F., Jiskoot W. (2013). Analytical approaches to assess the degradation of therapeutic proteins. TrAC Trends Anal. Chem..

[182] Lewnard J.A., Cobey S. (2018). Immune History and Influenza Vaccine Effectiveness. Vaccines.

[183] CDC CDC Seasonal Flu Vaccine Effectiveness Studies. https://www.cdc.gov/flu/vaccines-work/effectiveness-studies.htm.

[184] Sullivan S.G., Price O.H., Regan A.K. (2019). Burden, effectiveness and safety of influenza vaccines in elderly, paediatric and pregnant populations. Ther. Adv. Vaccines Immunother..

[185] Dhakal S., Klein S.L. (2019). Host Factors Impact Vaccine Efficacy: Implications for Seasonal and Universal Influenza Vaccine Programs. J. Virol..

[186] DiazGranados C.A., Dunning A.J., Kimmel M., Kirby D., Treanor J., Collins A., Pollak R., Christoff J., Earl J., Landolfi V. (2014). Efficacy of High-Dose versus Standard-Dose Influenza Vaccine in Older Adults. N. Engl. J. Med..

[187] Arnou R., Icardi G., De Decker M., Ambrozaitis A., Kazek M.P., Weber F., Van Damme P. (2009). Intradermal influenza vaccine for older adults: A randomized controlled multicenter phase III study. Vaccine.

